# NMR crystallography reveals active-site protonation states of Toho-1 β-lactamase in complex with avibactam

**DOI:** 10.1073/pnas.2616547123

**Published:** 2026-09-15

**Authors:** Christopher G. Williams, Songlin Wang, Veronica Carta, Patricia S. Langan, Alexander F. Thome, Sebastian A. Ramos, Jacob B. Holmes, Rittik K. Ghosh, Kevin L. Weiss, Gregory J. O. Beran, Joshua D. Hartman, Leighton Coates, Chad M. Rienstra, Leonard J. Mueller

**Affiliations:** ^a^Department of Chemistry, University of California-Riverside, Riverside, CA 92521; ^b^ Department of Biochemistry, University of Wisconsin-Madison, Madison, WI 53706; ^c^ National Magnetic Resonance Facility at Madison, University of Wisconsin-Madison, Madison, WI 53706; ^d^ Oak Ridge National Laboratory, Neutron Scattering Science Division, Oak Ridge, TN 37831

**Keywords:** Toho-1 β-lactamase, avibactam, NMR crystallography, MAS CryoProbe, UMA MLIP

## Abstract

The determination of active-site protonation states is critical for a full mechanistic understanding of enzyme catalysis and inhibition. Here, we employ NMR crystallography—the integrated combination of solid-state NMR spectroscopy, X-ray diffraction, and first-principles computational chemistry—to determine the protonation states of the active site of Toho-1 β-lactamase in complex with the non-β-lactam inhibitor avibactam. We report two X-ray crystal structures of the Toho-1:avibactam complex, along with high-field solid-state NMR measurements that enable near-complete backbone and side-chain resonance assignments. To overcome the computational scaling limits that have traditionally hindered NMR crystallography in large systems, we use an accelerated workflow in which machine-learning interatomic potentials enable efficient geometry refinement prior to density functional theory chemical shift calculations. For Toho-1, quantitative analysis of the active-site chemical shifts and chemical shift tensors using this hybrid protocol reveals that the key active-site side chains retain their canonical charge states in the presence of avibactam, with Lys73 and Lys234 protonated and positively charged, and Glu166 deprotonated and poised to function as a general base. Contrary to recent proposals suggesting that avibactam inhibits by suppressing essential proton transfers through p*K*_a_ perturbations, our data point to a more direct chemical origin arising from the intrinsic resistance of the Ser70-avibactam carbamoyl linkage to hydrolysis.

## Significance

Understanding enzyme mechanisms requires precise knowledge of active-site protonation states—features largely inaccessible to conventional structural biology. Here, we introduce an accelerated NMR crystallography workflow that makes practical the characterization of enzyme complexes at this fine level of chemical detail. This scalable approach utilizes machine-learning interatomic potentials for geometry refinement, bypassing a major computational bottleneck, while retaining high-accuracy density functional theory for chemical shift calculations. Our study of a clinically relevant β-lactamase:inhibitor complex identifies a protonation state configuration that challenges prevailing mechanistic models. These findings demonstrate the power of NMRX to resolve the chemical features essential to biological function and drug design, refining our understanding of how inhibitors bypass bacterial defenses.

β-Lactam antibiotics target penicillin-binding proteins in the bacterial membrane, inhibiting peptidoglycan biosynthesis and ultimately leading to bacterial cell death. To counter these potent antimicrobials, bacteria have evolved β-lactamases, enzymes that inactivate β-lactam antibiotics by hydrolyzing the amide bond of the β-lactam ring (1, 2). These enzymes are grouped into four classes: the Class B metalloenzymes and the serine-hydrolase Classes A, C, and D (3). Among the latter, Class A extended-spectrum β-lactamases (ESBLs) represent a particularly acute threat due to their broad substrate profiles (1, 4, 5). ESBLs hydrolyze an increasingly wide range of β-lactam antibiotics, including third-generation cephalosporins, and are now among the most widespread β-lactamases worldwide (6–9). Within this class, Toho-1 β-lactamase (CTX-M-44) is notable for its high catalytic activity against extended-spectrum cephams (4, 10).

**Fig. 1.**
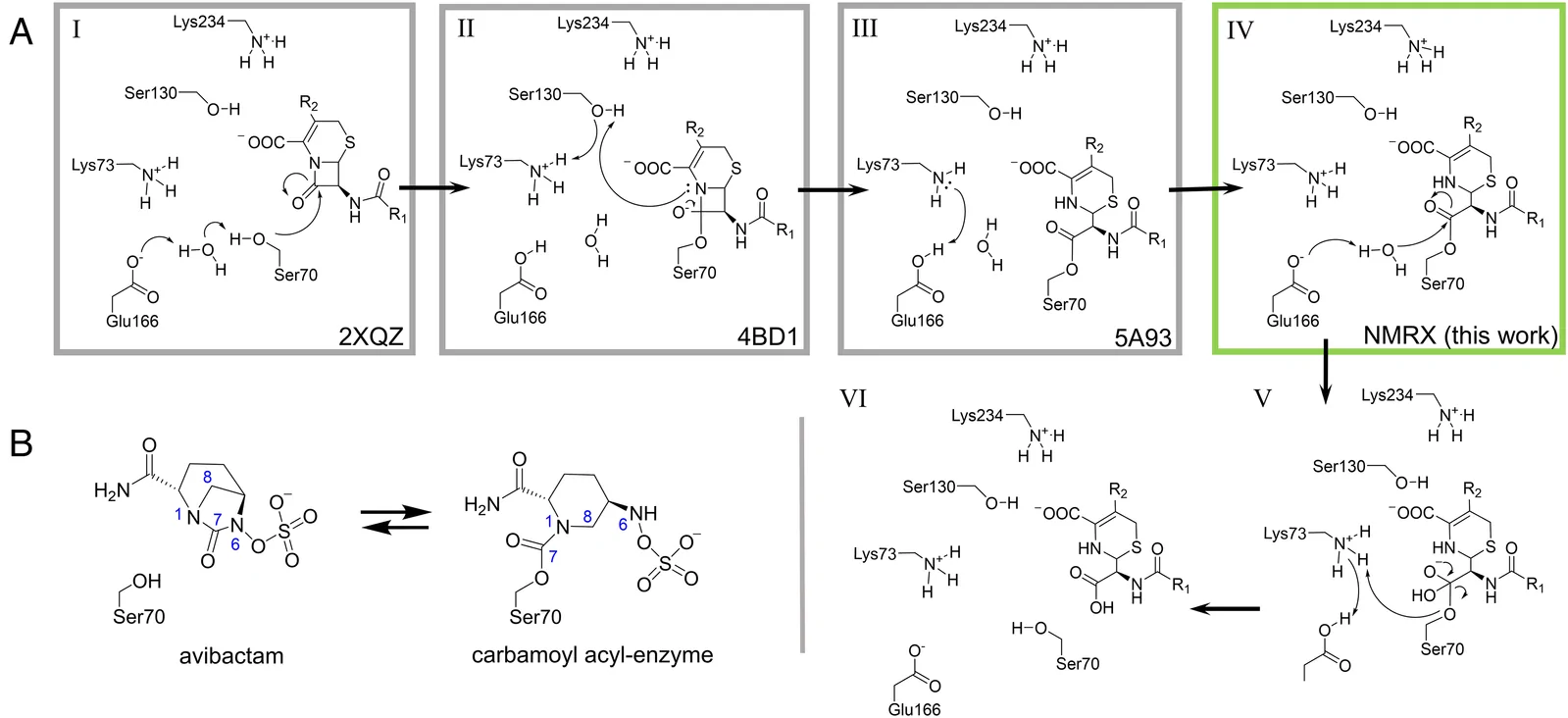
Catalytic mechanism of Class A serine β-lactamases. (*A*) General mechanism for the hydrolysis of β-lactam substrates by Class A serine β-lactamases, highlighting intermediates with analogs whose protonation states have been determined via neutron diffraction (gray boxes; PDB IDs indicated) and NMRX (green box; this work). (*B*) Avibactam forms a covalent carbamoyl linkage with Ser70 that mimics the acyl-enzyme intermediate (IV) but does not undergo hydrolytic dissociation; rather, slow decarbamylation triggers recyclization of the ring and regenerates the intact inhibitor.

Class A serine β-lactamases hydrolyze β-lactam antibiotics through a two-stage mechanism involving covalent acylation followed by hydrolytic deacylation (Fig. 1*A*) (3). The side chain of Ser70 initiates the reaction by nucleophilic attack on the β-lactam carbonyl to form an acyl-enzyme intermediate; deacylation then proceeds via a water molecule activated by general base catalysis. Central to this mechanism is the proton transfer network composed of the conserved residues Lys73, Glu166, and Ser130, with the latter positioned to mediate hydrogen bonding and proton shuttling (11). Lys234, though more distal, has also been implicated in modulating electrostatics and may contribute to alternative proton transfer pathways (12–15).

To combat the deactivation of β-lactam antibiotics, they are often coadministered with mechanism-based inhibitors, such as clavulanic acid, sulbactam, and tazobactam (16, 17). These inhibitors form covalent acyl-enzyme intermediates with the catalytic Ser70 side chain. However, the β-lactam cores of these inhibitors can themselves be hydrolyzed by a growing number of resistant Class A β-lactamases, regenerating the catalytically active enzyme (18). The emergence of these resistant variants has driven the development of inhibitors that are structurally distinct from β-lactams. Avibactam, a diazabicycloalkane, exemplifies this strategy: it forms a covalent carbamoyl linkage with Ser70 that mimics the acyl-enzyme intermediate but does not undergo hydrolytic cleavage (19–21). Instead, decarbamylation triggers a recyclization of the ring, restoring the intact inhibitor capable of further rounds of inhibition (Fig. 1*B*).

Proton transfer networks and the protonation states of the catalytic residues are critical to the mechanism of Class A β-lactamases, dictating both the reaction pathway and the stability of the enzyme–inhibitor complex (11). Neutron crystallography has provided atomic-level insight into the protonation states across the early stages of catalysis. In Toho-1, three neutron structures have captured hydrogen atom positions in the resting enzyme (2XQZ), an analog of the tetrahedral intermediate (4BD1), and the first step in the formation of the acyl-enzyme intermediate (5A93); these states are highlighted in Fig. 1*A* (22–24). These chemically detailed structures support a mechanism in which Glu166 and Lys73 act as alternating general acid–base catalysts. However, the protonation states in the inhibited form—specifically in the long-lived covalent complex with avibactam—remain unresolved. Previous mechanistic models, most notably one based on the ultrahigh-resolution crystal structure of CTX-M-14 (0.83 Å), have proposed that avibactam suppresses the Lys73-Glu166 proton transfer (Step III to IV in Fig. 1*A*) by perturbing the p*K_a_* of Lys73, thereby stabilizing an inactive state in which both Lys73 and Glu166 are neutral (25, 26). Although several additional high-resolution X-ray crystal structures of avibactam-bound class A β-lactamases have been reported, these complexes have so far resisted characterization by neutron crystallography (25–27).

Here, we employ NMR crystallography (NMRX)—the integrated application of X-ray diffraction, solid-state NMR (SSNMR), and first-principles computational chemistry—to resolve the active-site protonation states of the avibactam-inhibited Toho-1 complex. Although originally developed in the context of molecular organic and inorganic solids (28–33), the application of NMRX to biomolecular systems has advanced rapidly (34–42), driven in part by improvements in computational methods and the increasing accuracy of first-principles chemical shift prediction, even for large protein systems (43–46).

To this end, we report two X-ray crystal structures of the Toho-1:avibactam complex. These are complemented by high-field and high-sensitivity cryogenic CPMAS SSNMR experiments that enable near-complete backbone and side-chain chemical shift assignments. To quantitatively interpret these data, we use a hybrid machine-learning interatomic potential (MLIP)/density functional theory (DFT) protocol in which MLIP provides rapid geometry refinement, while DFT ensures high-accuracy chemical shift calculations. This scalable framework accelerates the geometry refinement step by five orders-of-magnitude relative to our previous DFT-based protocols (38, 41), reducing computational wall times from weeks to seconds, while maintaining the structural precision required for rigorous chemical shift prediction and quantitative discrimination among candidate structural models (47).

NMRX reveals that both Lys73 and Glu166 retain their canonical charge states in the avibactam-inhibited form of Toho-1. Contrary to recent proposals suggesting that avibactam inhibits by suppressing essential proton transfers through p*K*_a_ perturbations, our data point to a more direct chemical origin rooted in the intrinsic resistance of the Ser70-avibactam carbamoyl linkage to hydrolysis (19, 26, 48, 49). This revised mechanism refines the current understanding of avibactam action and highlights the power of NMRX to resolve critical protonation states that remain inaccessible to conventional structural biology.

## X-Ray Crystallography: Toho-1 Structures Display Conserved Avibactam Binding Features.

**Fig. 2.**
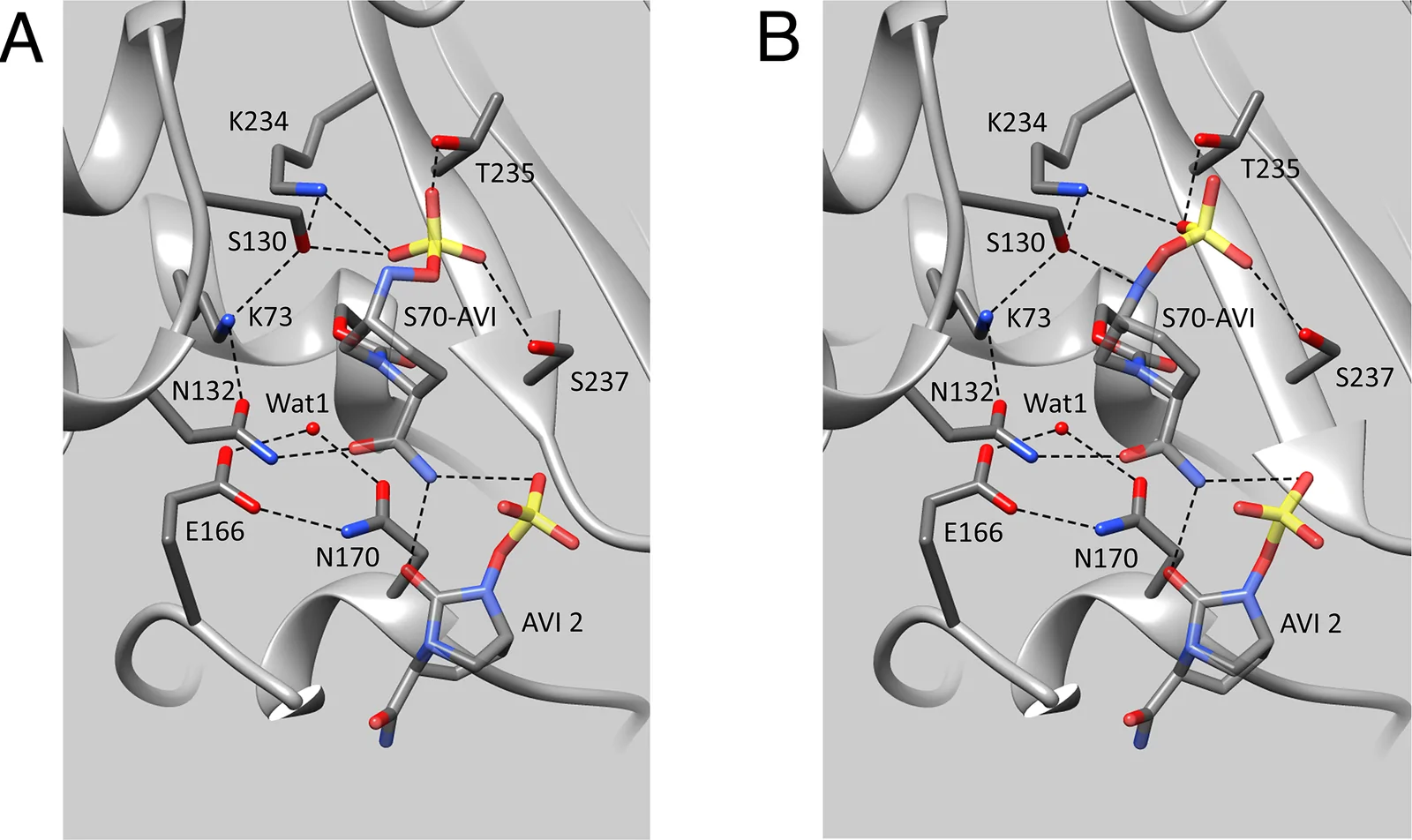
X-ray crystal structures of Toho-1 β-lactamase (R274N/R276N double mutant) in complex with avibactam. (*A*) 1.15 Å resolution structure solved at pH 6.1 under high salt conditions (2 M ammonium sulfate; PDB ID 9ZO2). (*B*) 2.0 Å resolution structure solved at pH 6.5 under low salt conditions (20 mM MES; PDB ID 9ZQQ). Both active sites display the covalent Ser70-avibactam carbamoyl linkage and a second, non-covalently bound avibactam. Hydrogen bonds are indicated by the dashed lines. Glu166 and Asn170 coordinate the catalytically essential water, positioned ~3.0 Å from the carbamoyl carbon. The two structures are nearly superimposable, with the exception of the avibactam sulfate group, which adopts distinct rotameric states that alter its interactions with Ser130: in (*A*) Ser130 hydrogen bonds to the sulfate, whereas in (*B*) Ser130 interacts with the avibactam N6.

X-ray crystal structures of Class A β-lactamases bound to avibactam have been reported for several enzymes, including CTX-M-14, CTX-M-15, and KPC-2 (25, 26, 49); however, no structures of the Toho-1:avibactam complex have previously been determined. Here, we report the X-ray crystal structure of Toho-1 in complex with avibactam solved at 1.15 Å resolution (PDB ID 9ZO2). In this structure, avibactam forms the characteristic covalent carbamoyl linkage to the nucleophilic Ser70, generating the acyl-enzyme intermediate analog, as shown in Fig. 2*A*. The active-site interactions with avibactam closely mirror those observed in other CTX-M complexes (25, 26): the carbamoyl carbonyl oxygen occupies the oxyanion hole formed by the backbone amides of Ser70 and Ser237; the sulfate lies within hydrogen-bonding distance to the sidechains of Ser130, Thr235, and (weakly) Lys234 and Ser237; and the carboxamide group engages in hydrogen bonding with the side chain of Asn132. Notably, in the Toho-1:avibactam structure, the carboxamide group also forms hydrogen bonds to the sulfate and carbonyl of a second, noncovalently bound avibactam at the entrance to the active site. This second avibactam has not been observed in other CTX-M crystal structures, but it is consistent with our previous solution-state NMR chemical shift perturbations for Toho-1 upon avibactam binding (50).

As in previous avibactam structures, the side chain of Glu166 interacts with the carboxamide of Asn170, and together they coordinate the catalytically essential water, which sits 3.0 Å from the avibactam carbamoyl carbon. The other active-site side chains form an extended hydrogen bonding network proximal to the bound inhibitor, linking Lys234, Ser130, Lys73, and Asn132. Ser130 further participates in a hydrogen bond with the avibactam sulfate—an interaction also observed in the pH 5.3 CTX-M-14:avibactam complex (26). Pemberton et al. proposed that this Ser130–avibactam hydrogen bond serves as a marker for the protonation state of Lys73: a charged Lys73 donates a hydrogen bond to Ser130, which in turn donates a hydrogen bond to the sulfate group (26). Under this model, the Ser130–avibactam interactions observed in both the Toho-1 and pH 5.3 CTX-M-14 complexes are consistent with a charged Lys73 side chain.

We also prepared a second crystal form of the Toho-1:avibactam complex under low-salt conditions (20 mM MES, pH 6.5) compatible with SSNMR, and solved its structure at 2.0 Å resolution (PDB ID 9ZQQ, Fig. 2*B*). As shown in Fig. 2 and *SI Appendix*, Fig. S9*A*, the protein backbone and active site of the two structures are nearly superimposable, with one notable exception: in the 2.0 Å structure, the conformationally flexible sulfate group adopts a different rotameric state that places the Ser130 β-hydroxyl within hydrogen bonding distance to the avibactam N6 nitrogen. This conformation is equivalent to the major conformer observed for the pH 7.9 CTX-M-14:avibactam complex also reported by Pemberton et al (26). We note that the Ser130 β-hydroxyl and avibactam N6 can both act as hydrogen bond donors or acceptors, leaving the directionality of the interaction—and therefore its implications for the protonation state of Lys73—ambiguous.

## Solid-State NMR: Probing the Chemistry of the Active Site.

Polycrystalline samples for SSNMR were prepared under the same low-salt batch crystallization conditions and pH (6.5) as the 2.0 Å X-ray structure, enabling the direct integration of the NMR and crystallographic data. SSNMR experiments were performed at 900 MHz, leveraging recent advances in probe, receiver, lock, and decoupling performance (51). These measurements were supplemented by high-sensitivity experiments performed at 600 MHz using a cryogenically cooled CPMAS probe, in which the coil and detection electronics are cooled to cryogenic temperatures (~20 K), while the sample remains at ambient temperature (52).

Chemical shifts of the active-site residues were determined using an “outside-in” approach, in which the full backbone and then the side-chain resonances were assigned for a uniformly-^13^C,^15^N-labeled sample at 900 MHz. The backbone assignments followed from our previous assignments of the inhibitor-free form (53). Side-chain resonances were then assigned using a combination of 3D heteronuclear NCACX experiments and 2D and 3D ^13^C homonuclear correlation experiments. Full assignment details are provided in *SI Appendix*.

**Fig. 3.**
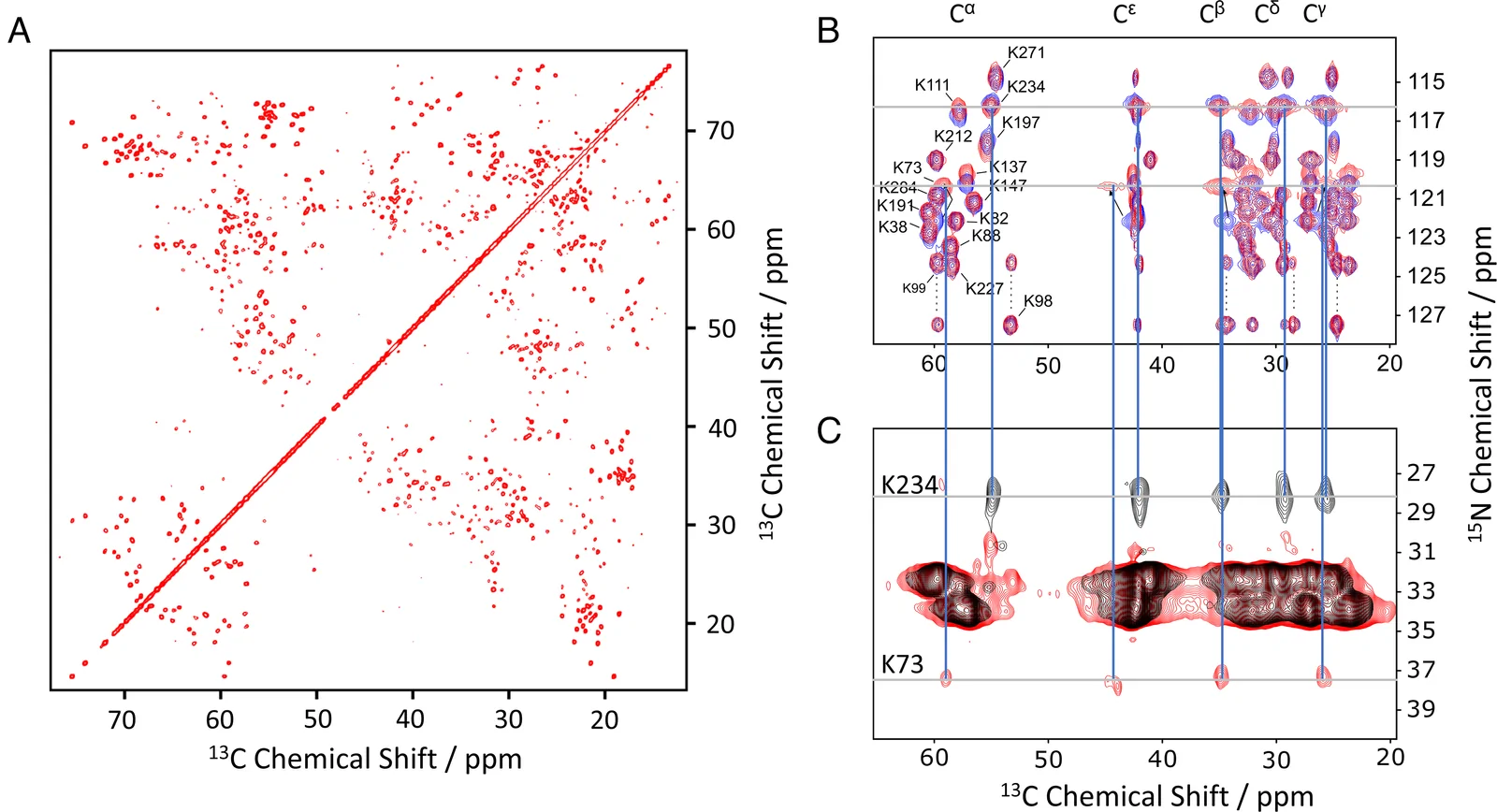
Solid-state NMR spectra and resonance assignments for the 28.5 kDa (263 amino acid) Toho-1:avibactam complex. (*A*) ^13^C–^13^C chemical shift correlation showing well-resolved cross peaks across the aliphatic region; spectra acquired at 900 MHz, 40 kHz MAS, and 50 ms DARR mixing (54). (*B*) NcaCX correlation spectra of residue-specific U-^13^C,^15^N-Lys labeled Toho-1 (blue) and the Toho-1:avibactam complex (red). (*C*) The NZCX correlation spectrum of the U-^13^C,^15^N-Lys labeled Toho-1:avibactam complex, enabling the unambiguous assignment of the Lys73 (red, 268 K) and Lys234 (black, 288 K) N^ζ^ chemical shifts. Spectra in (*B)* and (*C*) were acquired at 600 MHz on a Bruker CPMAS CryoProbe under 13.5 kHz MAS, utilizing 4 ms SPECIFIC CP (55) and 200 ms DARR mixing as described in the expanded *SI Appendix*, *Materials and Methods*.

Despite the high resolution achieved for these well-ordered crystalline samples, assignment of lysine N^ζ^ resonances for the 16 lysine residues in Toho-1 remained challenging due to the limited chemical shift dispersion of both N^ζ^ and the side-chain carbons. To address this, we prepared a second sample utilizing residue-specific ^13^C,^15^N labeling of the lysine residues for experiments on a CPMAS CryoProbe at 600 MHz. The combination of residue-specific labeling and the high sensitivity of the cryoprobe enabled unambiguous assignment of the lysine side chains: NcaCX spectra (Fig. 3*B*) displayed well-resolved peaks tracing the full carbon side chains, while 2D NZCX correlation experiments (Fig. 3*C*) provided clear N^ζ^ assignments for the active site Lys234 and Lys73 based on their distinct side-chain chemical shifts; 3D NCANZ experiments (*SI Appendix*, Fig. S7) allowed the assignment of the remaining N^ζ^ shifts. In total, 93% (1,463/1,572) of all carbon and nitrogen atoms were assigned, including 99.0% (772/780) of backbone atoms and 90.6% (637/703) of side-chain carbon atoms (*SI Appendix*, Fig. S11).

This comprehensive dataset provides direct access to side-chain chemical shifts for the key active-site residues Ser70, Lys73, Ser130, Asn132, Glu166, Asn170, and Lys234, as summarized in Table 1. Relative to the free enzyme, the covalent complex shows characteristic chemical shift perturbations indicative of avibactam binding, with the Ser70 amide nitrogen shifting downfield from 118.9 to 121.8 ppm, C^α^ shifting upfield from 62.7 to 60.9 ppm, and the C^β^ resonance, absent in both solution- and solid-state spectra of the free enzyme, appearing at 67.6 ppm. Across the protein, chemical shift perturbations are concentrated within and at the entrance to the active site (*SI Appendix*, Fig. S12), consistent with the presence of both the covalent and noncovalent avibactam molecules observed crystallographically.

**Table 1.** Key experimental and first-principles isotropic chemical shifts and tensor principal components (PC) for the four best-fit models, reported in ppm

Residue	Atom	Expt	Model 010	Model 101	Model 110	Model 111	Residue	PC	Expt	Model 010	Model 101	Model 110	Model 111
Ser70	C^β^	67.6	68.8	66.7	67.4	67.9	Lys73	δ_11_	50.1	49.7	57.9	48.3	46.5
Lys73	C^γ^	25.6	24.6	25.3	24.3	24.5	N^ζ^	δ_22_	39.0	37.1	19.5	38.1	38.1
	C^δ^	31.5	31.6	35.8	32.4	31.0		δ_33_	23.4	25.8	17.2	24.4	23.8
	C^ε^	43.5	43.6	45.4	43.9	44.5	Glu166	δ_11_	246.1	248.0	264.2	247.9	265.5
	N^ζ^	37.5	37.5	31.5	36.9	36.2	C^δ^	δ_22_	179.4	187.0	177.3	188.0	176.0
Ser130	C^β^	62.7	62.2	61.9	62.6	63.4		δ_33_	111.9	106.3	104.5	106.4	105.2
Asn132	C^γ^	172.3	172.5	172.5	172.7	175.7	Lys234	δ_11_	41.9	34.5	39.1	39.9	37.8
	N^δ^	109.9	110.6	112.1	111.6	118.7	N^ζ^	δ_22_	28.3	7.1	23.1	22.8	21.6
Glu166	C^γ^	30.5	33.4	30.3	33.2	30.2		δ_33_	15.0	1.9	17.1	17.8	18.6
	C^δ^	179.1	180.4	182.0	180.7	182.2	tensor PC		red-*χ*^2^	1.77	3.35	0.76	2.88
Asn170	C^γ^	176.8	174.3	175.7	174.6	176.4							
	N^δ^	118	118.5	118.3	118.5	118.4							
Lys234	C^ε^	42.4	40.2	41.3	41.4	41.8							
	N^ζ^	28.4	14.5	26.4	26.8	26.0	Isotropic + tensor			010	101	110	111
isotropic		red-*χ*^2^	2.24	2.22	1.10	1.83	Composite	red-*χ*^2^		2.06	2.66	0.97	2.24

The side-chain shifts also provide residue-specific probes that allow an initial assessment of the active-site protonation states. The Lys73 N^ζ^ resonance at 37.5 ppm indicates a protonated, positively charged side-chain ammonium group, challenging the previous neutral-state model (26). Notably, all side-chain carbon resonances for Lys73 are broadened relative to those of the other lysine residues (Fig. 3*B*), and ^15^N exchange spectroscopy experiments (EXSY; *SI Appendix*, Fig. S10) reveal exchange with a second state with an N^ζ^ shift of 33.8 ppm. This second N^ζ^ shift remains consistent with a protonated lysine, indicating local conformational exchange rather than a change in charge state. The Lys234 chemical shifts are also most consistent with a protonated, positively charged side chain, although its N^ζ^ resonance at 28.4 ppm lies slightly below the typical range of 29 to 36 ppm for a charged lysine (56–58), leaving some ambiguity. In contrast to Lys73, the side-chain carbon resonances of Lys234 are sharp and well-resolved, while its N^ζ^ resonance is broadened and narrows with increasing temperature (*SI Appendix*, Fig. S10*B*). This temperature dependence suggests local dynamics near the avibactam sulfate group, to which Lys234 serves as a weak hydrogen-bond donor.

For Glu166, the interpretation of the side chain chemical shifts is complicated by the local hydrogen-bonding environment. At 179.1 ppm, the C^δ^ resonance lies between values expected for charged (~182 ppm) and neutral (~177 ppm) glutamate residues (56). This intermediate shift likely reflects a strong hydrogen bond to the conserved active-site water, which can shift the resonance of a charged glutamate upfield or a neutral glutamate downfield.

## NMR Crystallography: Resolving Active-Site Protonation States with a Hybrid MLIP/DFT Approach.

To rigorously interpret the active-site chemical shifts, we integrate the structural and spectroscopic data above with computational chemistry. Rather than relying on empirical correlations for individual shifts, we use an NMRX protocol that evaluates complete three-dimensional active-site models by quantitatively comparing their predicted chemical shifts with experiment. Our workflow follows our previously established NMRX protocols (38, 41) with one key modification: FAIRChem’s Universal Model for Atoms (UMA) (59) is incorporated for rapid MLIP geometry refinement, while DFT is retained for high-accuracy chemical shielding calculations. This replaces the rate-limiting DFT relaxation step, reducing the computational cost of geometry optimization by a factor of ~10^5^ while preserving ^13^C and ^15^N chemical shift prediction accuracy relative to our previous DFT-refined workflow, as demonstrated by benchmarking on organic crystals described in *SI Appendix* and in recent independent work (60).

**Fig. 4.**
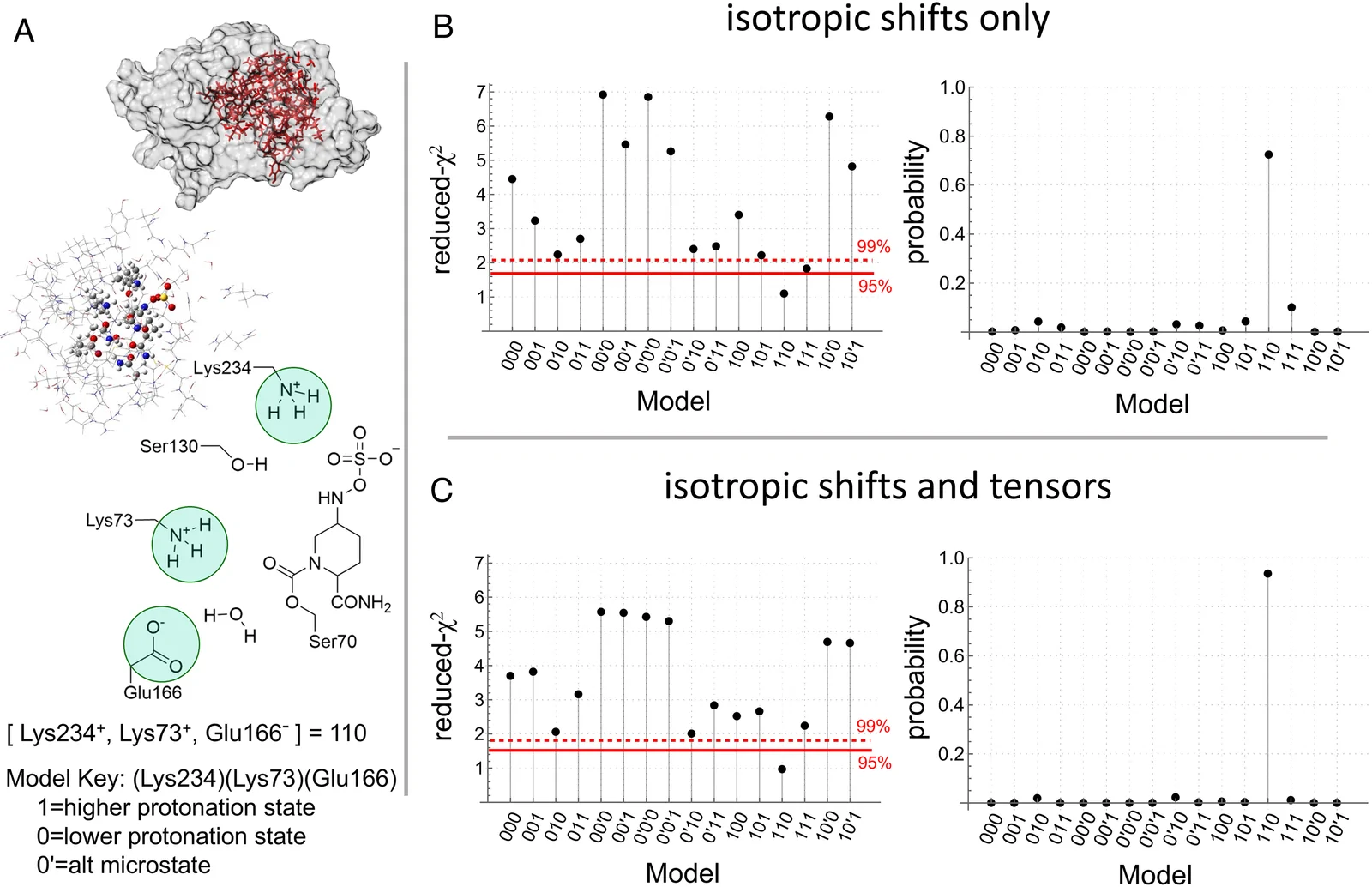
NMR Crystallography of the Toho-1:avibactam complex. (*A*) Active-site cluster construction: (*Top*) X-ray framework (PDB ID 9ZQQ) with the active site highlighted in red; (*Middle*) cluster model used for UMA MLIP geometry optimization and DFT chemical shift calculations; (*Bottom*) Detail of the mechanistically critical residues Lys234, Lys73, and Glu166, where protonation states were systematically varied. (*B* and *C*) Statistical ranking of candidate models against experimental ^13^C and ^15^N shifts using the reduced-*χ*^2^ statistic (95% and 99% confidence limits shown as the solid and dashed red horizontal lines, respectively) along with UC Model probabilities. (*B*) Isotropic shift analysis: Comparison based solely on the isotropic chemical shifts does not provide a unique basis to select between the canonical [K234^+^, K73^+^, E166^−^] and the fully-protonated [K234^+^, K73^+^, E166^0^] charge states. (*C*) Composite shift analysis: Inclusion of both isotropic and chemical shift tensor principal components improves discrimination between the candidate models, and identifies with high probability (>93%) the canonical protonation state model, [K234^+^, K73^+^, E166^−^], as the experimental structure.

To systematically explore the active-site protonation states, 16 candidate structures were generated by varying the protonation states and hydrogen atom positions for Lys234, Lys73, and Glu166, as detailed in *SI Appendix*. Each model was built from the 2.0 Å X-ray Toho-1:avibactam structure (Fig. 4) and geometry optimized using the MLIP-based workflow. DFT chemical shift calculations with locally dense basis sets (44, 61) were then performed for each optimized structure, and the resulting ^13^C and ^15^N chemical shifts were compared to experiment using the reduced-*χ*^2^ statistic (Table 1 and *SI Appendix*, Table S6). Full computational details are provided in *SI Appendix*.

As summarized in Fig. 4*B*, the best-fit model has all three of the ionizable active-site residues in their canonical protonation states: the side chains of Lys234 and Lys73 are both protonated and positively charged, while that of Glu166 is deprotonated and negatively charged. We designate this state as [K234^+^, K73^+^, E166^−^], where the superscripts denote the charge of each residue and the ordering follows the geometric arrangement of the active-site residues as viewed from the perspective in Fig. 2.

Although the canonical protonation state model provides the best agreement with the experimental shifts, it is not uniquely supported: a second model, [K234^+^, K73^+^, E166^0^], in which Glu166 is protonated and neutral, falls near the 95% confidence limit of the reduced-χ^2^ statistic. This fully protonated active-site model lacks a general base to activate the catalytic water for hydrolytic deacylation. This model shares a key mechanistic feature with the previous hypothesis of Pemberton et al., in which avibactam inhibition was proposed to arise from stabilization of a neutral Glu166 state (26).

To evaluate the relative support for these candidate models, the posterior probability that each represents the correct experimental structure was quantified using the Uniform chi-squared (UC) model, a hierarchical Bayesian approach (62–64). As shown in Fig. 4*B*, both the canonical [K234^+^, K73^+^, E166^−^] model and the fully-protonated [K234^+^, K73^+^, E166^0^] model have nonnegligible UC model probabilities, 74% and 10%, respectively. The UC Model probabilities favor a deprotonated Glu166 but do not exclude the neutral state as a plausible alternative.

**Fig. 5.**
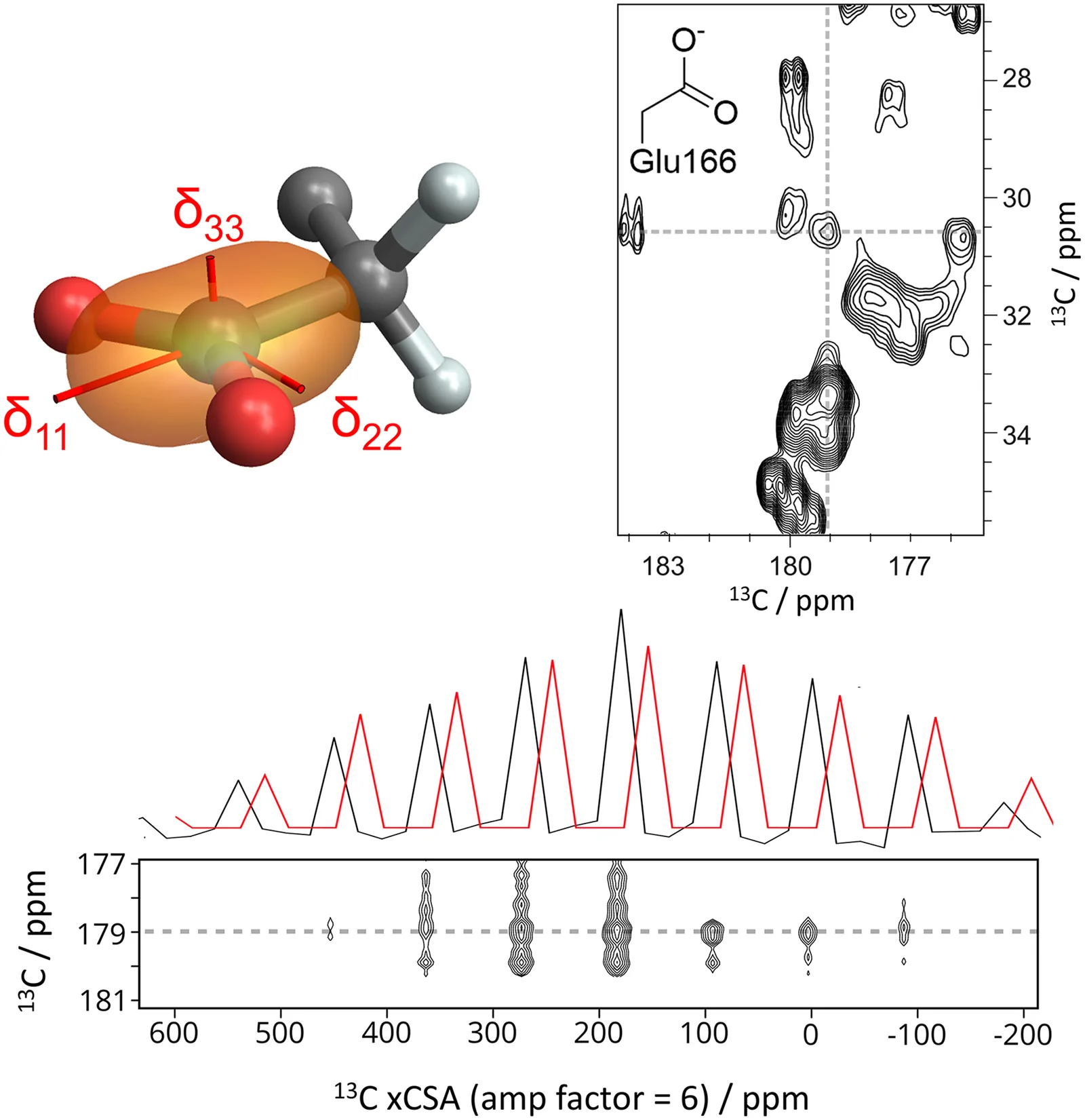
Glu166 C^δ^ chemical shift tensor. (*Top*) The Glu166 C^γ^–C^δ^ cross peak is well resolved in the 2D CC DARR spectrum, permitting the measurement of the C^δ^ chemical shift tensor using a 3D xCSA experiment (65, 66). (*Bottom*) The best-fit simulation (red) to the experimental sideband manifold extracted from the 2D plane yields the CSA principal axis components (δ_11_, δ_22_, δ_33_) = (246.1±1.6, 179.4±1.2, 111.9±1.4) ppm. Molecule and tensor rendered in TensorView (69).

We therefore turned to chemical shift tensors as additional probes of the Glu166, Lys73, and Lys234 protonation states. The Glu166 C^γ^-C^δ^ cross peak is well resolved in the 2D CC DARR spectrum, enabling acquisition of the C^δ^ chemical shift tensor using a 3D version of the chemical shift amplification (xCSA) experiment (Fig. 5) (65, 66). The resulting tensor components (Table 1) closely match those observed empirically for a charged carboxylate group and are inconsistent with the significantly larger span expected for a neutral carboxylic acid (67). Similarly, the N^ζ^ resonances of Lys73 and Lys234 are well resolved in 1D spectra, permitting measurement of their ^15^N chemical shift tensors using a 2D xCSA experiment (*SI Appendix*, Fig. S13). For both active-site lysines, the observed tensor components (Table 1) are in excellent agreement with the expected values for a protonated, positively charged ammonium group and are inconsistent with the larger spans characteristic of a neutral amine (68).

Combining the isotropic chemical shifts and tensor principal components into a composite reduced-χ^2^ sharply improves the discrimination between the candidate models (Fig. 4*C* and Table 1). Under this combined analysis, the canonical protonation state model, [K234^+^, K73^+^, E166^−^], emerges with high confidence (>93%) as the experimental structure. In contrast, the alternative, fully-protonated state with neutral Glu166 is no longer statistically supported (<1%).

As an independent test, the ^1^H chemical shift of the active-site Lys73 H^ζ^ was measured using FSLG-HETCOR (*SI Appendix*, Fig. S14). ^1^H chemical shifts are particularly sensitive to hydrogen bonding and local electrostatics and have become valuable probes in NMR crystallography (40, 70–72). The observed Lys73 H^ζ^ shift of 7.2 ppm directly supports a protonated Lys73 ammonium group in the inhibited complex. For comparison, the corresponding DFT-predicted shifts are 6.3 ppm for the canonical [K234^+^, K73^+^, E166^−^] model and 0.9 ppm for the [K234^+^, K73^0^, E166^0^] model.

**Fig. 6.**
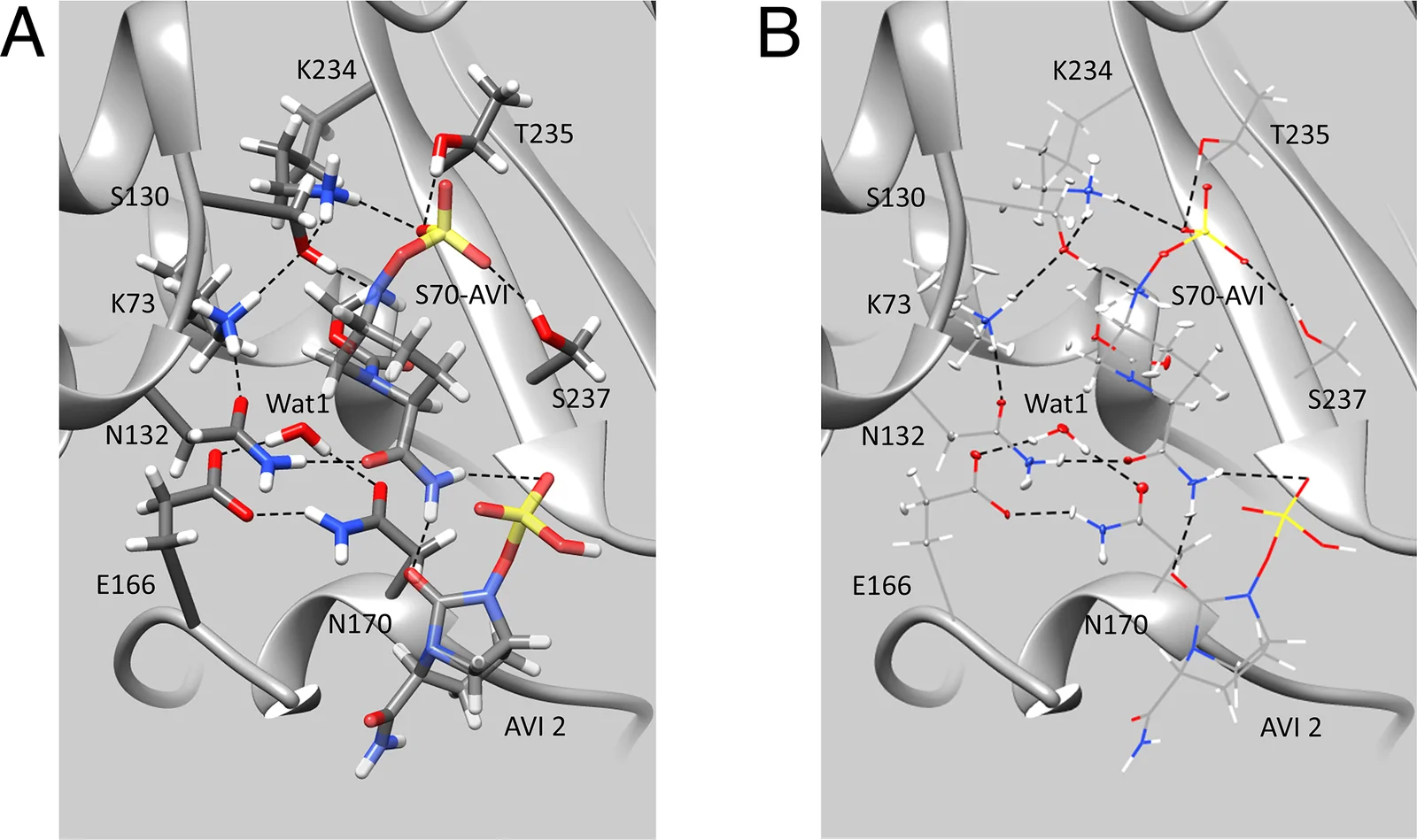
NMR crystal structure of the Toho-1:avibactam complex. (*A*) Active site with explicit protonation states in which Lys73, Glu166, and Lys234 adopt their canonical charged states. (*B*) Spatial precision of the NMRX-refined coordinates. ADPs are derived from the ensemble of structures with Bayesian UC Model probabilities ≥5%; ellipsoids depict the 95% coordinate confidence surface of the displacement distributions (scale factor = 2.8 σ).

Together, X-ray crystallography, SSNMR, and first-principles computational chemistry yield the NMR crystal structure of the Toho-1:avibactam complex shown in Fig. 6. In this structure, the positions of all active-site atoms, including hydrogens, are explicitly defined. The anisotropic displacement parameters (ADPs) shown in Fig. 6*B* are estimated using the Hofstetter-Emsley method (73), adapted for our cluster model approach and Bayesian UC probability analysis as detailed in *SI Appendix*. These uncertainties correspond to positional rmsds of 0.07 Å for heavy atoms and 0.09 Å for hydrogen atoms, comparable to those reported for other high-resolution NMR crystal structures (41, 73). Notably, there is no significant difference between the positional uncertainties for the active-site side chains and those for the covalently bound avibactam, even though only the amino acid side-chain chemical shifts were included in the reduced-χ^2^ analysis. In contrast, the ADPs for atoms at the periphery of the cluster are nearly twice as large. This is consistent with the local sensitivity of ^13^C and ^15^N chemical shifts to geometry within ~2 to 4 Å (44), with structural perturbations beyond this range having a diminishing impact. These results suggest that bound ligands proximal to the shift region can be refined with useful precision even when their chemical shifts are not directly measured.

## Mechanistic Implications: The Chemical Basis of Toho-1 Inhibition.

The NMR crystal structure of the Toho-1 β-lactamase:avibactam complex provides a chemically detailed snapshot of the inhibited state, including explicit active-site protonation states. In this canonical configuration, Lys73 and Lys234 are protonated and positively charged, while Glu166 is deprotonated and optimally positioned to function as a general base. The catalytically essential water is oriented for nucleophilic attack on the carbamoyl carbon, adopting the expected configuration just prior to hydrolytic deacylation. Yet hydrolysis does not occur, and the complex remains trapped in this long-lived inhibited state. This structure establishes that hydrolysis is not suppressed by a reconfiguration of the active-site protonation states. With the enzyme poised for reaction, the barrier must instead arise primarily from the chemistry of the inhibitor itself and the intrinsic resistance of the Ser70-avibactam carbamoyl linkage to hydrolysis. Carbamoyl linkages are fundamentally less reactive than the ester linkages formed with β-lactam substrates and inhibitors. Indeed, carbamates hydrolyze three to six orders-of-magnitude more slowly than the corresponding esters because of n→π* electron donation from the adjacent nitrogen, which significantly reduces the electrophilicity of the carbamoyl carbon (74–77). Prior studies have also pointed to the inherent stability of the carbamoyl linkage as a plausible origin of inhibition (11, 19, 48, 49), but the lack of direct experimental constraints meant that a reconfiguration of the active-site protonation states could not be ruled out.

This structure revises the current mechanistic understanding of Toho-1 inhibition by avibactam. It differs from recent proposals in which inhibition is driven by active-site p*K_a_* perturbations that neutralize Glu166 and Lys73 (26). That model is based on ultrahigh-resolution CTX-M-14:avibactam X-ray crystal structures together with solution-state NMR titration data on the related CTX-M-9:avibactam complex. In the latter, a pH-dependent C^ε^-H^ε^ cross peak was tentatively assigned to Lys73, and its chemical shift perturbation attributed to Lys73 deprotonation (p*K*_a_ < 6.5), although this NMR cross peak was never definitively assigned. As detailed in *SI Appendix*, we observe an analogous C^ε^-H^ε^ cross peak in the solution-state spectrum of Toho-1:avibactam with nearly identical chemical shifts and pH dependence; using our complete chemical shift assignments, however, we can unambiguously identify this resonance as Lys137 rather than Lys73 (*SI Appendix*, Fig. S8). If, as seems likely, the CTX-M-9 cross peak arises from the same conserved site, then the NMR evidence for a neutral Lys73 in the CTX-M-9 and CTX-M-14 inhibited complexes is called into question. By contrast, the protonation states for the Toho-1:avibactam complex are established directly from residue-specific solid-state NMR observables: the Lys73 N^ζ^ isotropic shift and tensor identify a protonated ammonium group, while the Glu166 C^δ^ shift tensor identifies a deprotonated carboxylate. NMRX allows us to quantitatively establish these charge states with a high level of statistical confidence.

The active sites of the pH 6.5 Toho-1:avibactam and pH 7.9 CTX-M-14:avibactam complexes are essentially superimposable (*SI Appendix*, Fig. S9*B*) and exhibit the same Ser130–N6 interaction, suggesting that they share the same protonation-state configuration. Establishing the generality of the canonical protonation-state configuration for inhibited complexes, however, will require direct measurements in CTX-M-14 and additional Class A β-lactamases. While neutron diffraction remains the gold standard for locating hydrogen atoms in structural biology, NMR crystallography offers an emerging complementary strategy for determining chemically detailed three-dimensional structures, including hydrogen atom positions, and resolving enzyme mechanisms at the atomic level.

## Conclusions

The NMR crystal structure of the Toho-1 β-lactamase:avibactam complex establishes that the active-site residues adopt their canonical protonation states: Lys73 and Lys234 are positively charged, while Glu166 is deprotonated and poised to function as a general base. The active site appears optimally configured for hydrolytic deacylation, with the catalytic water positioned for nucleophilic attack at the carbamoyl carbon. Yet hydrolysis does not occur. Instead, slow decarbamylation leads to regeneration of the intact inhibitor. Our findings demonstrate that avibactam’s inhibitory power does not stem from a reconfiguration of the active-site protonation states. Rather, inhibition is primarily a consequence of the chemistry of avibactam itself and the hydrolytic resistance of the Ser70-avibactam carbamoyl linkage. This stability underscores the broader role of carbamates as hydrolytically stable motifs in medicinal chemistry (77).

For Toho-1, neutron structures have previously established protonation states for the substrate-free enzyme, the tetrahedral intermediate, and the initial step in formation of the acyl-enzyme intermediate (Fig. 1*A*). The Toho-1:avibactam complex reported here provides a carbamoyl analog of the acyl-enzyme intermediate state immediately preceding hydrolytic deacylation. By integrating NMR crystallography with these previous neutron structures, we extend the experimentally grounded series of active-site protonation states to encompass a full analog of this mechanistically essential intermediate.

The inhibited Toho-1:avibactam complex has resisted characterization by neutron diffraction, as addition of avibactam has presented challenges for the required large crystals. By contrast, the microcrystals used for NMR crystallography are more robust to such perturbations, enabling chemically detailed characterization of the inhibited state that was not previously accessible. More broadly, these results underscore the unique value of NMR crystallography for capturing chemically rich structural detail and resolving enzyme mechanisms at the atomic level—including protonation states, hydrogen bonding, and local electrostatics—in challenging systems that remain inaccessible to the conventional tools of structural biology.

A practical limitation to NMR crystallography has been the computational expense of geometry optimization of candidate structures, which is a prerequisite for accurate chemical shift calculations. The emergence of general MLIPs that achieve DFT-level structural accuracy at a small fraction of the computational cost removes this major bottleneck. At present, protein NMRX still relies on first-principles DFT shielding calculations for model discrimination; for the 835-atom clusters used here, this requires modest resources, typically ~24 h wall time per structure on 32 CPU cores. However, rapid progress in machine-learning chemical shift prediction (78–82) suggests a path to further acceleration. Together, these advances point to the potential for increasingly global protein structural refinement through NMR crystallography.

## Materials and Methods

Toho-1 β-lactamase was expressed, purified, and crystallized in complex with avibactam following our earlier protocols (22, 24, 53), using the R274N/R276N double mutant to prevent aggregation in solution and crystal twinning (83). X-ray structures were determined under high-salt conditions (2 M ammonium sulfate; pH 6.1; 1.15 Å; PDB 9ZO2) and low-salt conditions compatible with SSNMR (20 mM MES; pH 6.5; 2.0 Å; PDB 9ZQQ). Solid-state NMR spectra were acquired at 900 MHz (1.6 mm HCN Black Fox Probe, 20 kHz MAS, 0 °C) and 600 MHz (3.2 mm HCN MAS CryoProbe, 13.5 kHz MAS, −5 °C). Computational models for NMR crystallography were based on active-site clusters built on the 9ZQQ framework; clusters included all residues and crystallographic waters within 8 Å of the avibactam ligand and contained 835 to 838 atoms, depending on the protonation model. Candidate models were geometry optimized using the UMA machine-learning interatomic potential (omol//uma-s-1p1) (59), with peripheral heavy atoms fixed at their crystallographic positions and all hydrogen atoms and internal heavy atoms allowed to relax. DFT chemical shieldings were calculated at the B3LYP level of theory in Gaussian16 (84), employing a three-tier, locally dense basis set (44, 61). Computed shieldings were converted to chemical shifts using benchmark-derived linear rescaling relationships based on established solid-state NMR test sets (44, 45). Candidate models were ranked by reduced-χ^2^ and Bayesian posterior analysis (63, 64) against the experimental ^13^C/^15^N isotropic shifts and tensor components (Table 1 and *SI Appendix*, Table S6). Detailed experimental parameters and computational/statistical methods are provided in *SI Appendix*.

## Supplementary Material

Appendix 01 (PDF)

Dataset S01 (TXT)

Dataset S02 (TXT)

## Data Availability

Crystallographic coordinates for the X-ray structures reported in this work have been deposited in the Protein Data Bank under accession codes 9ZOZ, 9ZQQ, and 9ZO2 (85–87). NMR chemical shift assignments for the Toho-1:avibactam complex have been deposited in the Biological Magnetic Resonance Data Bank under accession number 53206 (88). The NMRX-refined active-site coordinates for the 9ZQQ Toho-1:avibactam crystal structure, including the active-site hydrogen atoms and estimated ADPs, are provided as Dataset S1. Coordinates for all 16 UMA-optimized active-site cluster models are provided as Dataset S2. All other data are included in the manuscript and/or supporting information.
