# Supplementary material for: NMR crystallography reveals active-site protonation states of Toho-1 β-lactamase in complex with avibactam
Source: Proc Natl Acad Sci U S A. 2026 Sep 15;123(38):e2616547123. doi: 10.1073/pnas.2616547123 (PMC13598153; doi:10.1073/pnas.2616547123)
Supplement: Supplementary file 1 [file pnas.2616547123.sapp.pdf]

PNAS

Supporting Information for

NMR Crystallography Reveals Active-Site Protonation States of Toho-1 β-Lactamase in Complex with Avibactam

Christopher G. Williams[1], Songlin Wang[2,3], Veronica Carta[1], Patricia S. Langan[4], Alexander F. Thome[1], Sebastian A. Ramos,[1] Jacob B. Holmes[1], Rittik K. Ghosh[1], Kevin L. Weiss[4], Gregory J. O. Beran,[1] Joshua D. Hartman,[1] Leighton Coates[4], Chad M. Rienstra[2,3,5], Leonard J. Mueller[1]

[1]Department of Chemistry, University of California-Riverside, Riverside, CA 92521, USA
[2]Department of Biochemistry, University of Wisconsin-Madison, Madison, WI 53706, USA
[3]National Magnetic Resonance Facility at Madison, University of Wisconsin-Madison, Madison, WI 53706, USA
[4]Oak Ridge National Laboratory, Neutron Scattering Science Division, 1 Bethel Valley Rd, Oak Ridge, TN 37831, USA
[5]Current address: Resynant Inc., 8001 Terrace Ave., Middleton, WI 53562, USA

Corresponding Author information: Leonard J. Mueller

Email: leonard.mueller@ucr.edu

This PDF file includes:

Supplementary text:
- Computational Methods for NMR Crystallography S2
  - Figures S1-S3
- Backbone and Side-Chain Assignments from SSNMR S9
  - Figures S4-S7
- Assignment of the pH-Dependent Lysine Resonance to Lys137 S13
  - Figure S8
- Expanded Materials and Methods S15
  - Tables S1-S5

Figures S9-S14 S20
Table S6 S26
SI References S28

Other supporting materials for this manuscript include the following:

Supplementary Dataset S1: NMRX-refined active-site coordinates with explicit protonation states and estimated ADPs for the 9ZQQ Toho-1:avibactam crystal structure, in PDB format: Dataset_S1_9ZQQ_active_site_refined_NMRX.pdb.txt

Supplementary Dataset S2: Coordinates for all 16 UMA-optimized active-site cluster models: Dataset_S2_16_UMA_optimized_active_site_cluster_models.txt

Computational Methods for NMR Crystallography:

UMA-Optimized Geometries and First-Principles Chemical Shift Calculations:

Computations were performed using a hybrid MLIP/QM approach in which geometry optimizations were performed using a machine-learning interatomic potential (MLIP) and chemical shift calculations were performed using DFT. Incorporating MLIP accelerates geometry optimization of the cluster models below by five orders-of-magnitude compared to our earlier DFT-based protocol (7, 8). For the structures considered here, wall times decrease from 2-6 weeks per optimization on 32 CPU cores/64 threads to ~30 s on a GPU, while retaining the structural accuracy required for first-principles NMR chemical shift calculations. Benchmarking against the chemical shift test sets described below, as well as recent independent work (9), indicates that this replacement does not degrade prediction accuracy for ^{13}C, ^{15}N, and ^{1}H chemical shifts.

Generation of Clusters and Protonation-State Models: Computations were performed using a cluster-based model of the active site. The initial cluster was constructed from crystal structure 9ZQQ by selecting all atoms within 8 Å of Ser70, Lys73, Glu166, and the covalently bound avibactam, followed by expansion to include complete residues (Fig. S1). The resulting model was then processed as follows: (1) hydrogen atoms were added, (2) backbone N atoms at truncated N-termini were replaced by H, and (3) C-terminal carbonyls were capped as $-CONH_2$. The final models included residues Ala67-Ala78, Val103-Tyr105, Ala125-Leu127, Tyr129-Asn136, Leu138-Ile139, Phe160, Leu162, Arg164-Asn170, Asn214, Thr216-Gly217, Lys234-Gly238, Asp240, Asn245, Asn274, and Asn276; 17 crystallographic waters (9, 11, 18, 51, 57, 68, 90, 107, 118, 159, 192, 261, 282, 421, 485, 503, and 827); and both the covalently and non-covalently bound avibactam molecules. The total atom count varied from 835 to 838 atoms depending on the protonation state.

Within this cluster, candidate structures were systematically generated by varying the protonation states of the three ionizable side chains Lys234, Lys73, and Glu166 (Fig. S1C). Models were labeled using a three-position protonation index ordered Lys234-Lys73-Glu166, following the approximate geometric arrangement of the side chains in the active site from the perspective shown in Fig. S1C. For lysine residues, 1 denotes the protonated, positively charged ammonium state and 0 denotes the neutral amine state; for Glu166, 1 denotes the protonated, neutral carboxylic acid state and 0 denotes the deprotonated, negatively charged carboxylate state; for example, 101 corresponds to the model [$K234^+$, $K73^0$, $E166^0$], where the charge on each residue is given by the superscript.

Sixteen candidate models were considered, eight primary protonation-state combinations and eight alternative hydrogen bonding configurations. The three ionizable side chains give eight possible charge state combinations. When alternative proton placements are considered for neutral Lys234 and Lys73 (each of which can in principle be generated by removal of any one of three N^{ζ} protons) and for protonated Glu166 (for which either carboxylate oxygen can be protonated), these eight protonation states give rise to 48 possible proton-placement configurations. Retaining key crystallographic hydrogen bonds from Lys234 to the backbone carbonyl of Gly236, from Lys73 to the carboxamide carbonyl of Asn132, and from the Asn170 side chain amide to Glu166 reduces this set to 18. Requiring that Lys234 and Lys73 (whether protonated or neutral) maintain a hydrogen-bonding interaction with Ser130 eliminates two additional configurations, leaving the 16 candidate models considered here.

For neutral lysines, the model labels specify which N^{ζ} proton is removed. In the primary models (labeled with 0), Lys234 always donates a hydrogen bond to Ser130, while neutral Lys73 accepts a hydrogen bond from Ser130. In the alternative configurations (labeled with 0'), this arrangement is reversed: neutral Lys234 accepts a hydrogen bond from Ser130, and Lys73 always donates a hydrogen bond to Ser130. In both configurations, the avibactam N6-Ser130 hydrogen bond adjusts to maintain hydrogen bonding. Coordinates for all 16 geometry-optimized candidate models are provided as Supplementary Dataset S2.

UMA Geometry Optimization: All cluster models were geometry optimized using FAIRChem's Universal Model for Atoms (UMA) (omol//uma-s-1p1) in ASE with the LBFGS optimizer and the FAIRChemCalculator (10, 11). A two-tier protocol was employed, with the "high layer" comprising the side chains of Ser70 (+C^{α}), Lys73, Ser130 (+C^{α}), Asn132, Glu166, Asn170, and Lys234, together with the catalytically essential water (Wat1) and covalently bound avibactam (Fig. S1B). During optimization, high-layer heavy atoms and all hydrogen atoms were allowed to relax, while all other heavy atoms were fixed at their crystallographic coordinates. To anchor the model to the experimental electron density, the Wat1 oxygen and the sulfur atom of the avibactam sulfate group were held fixed at their crystallographic positions. Convergence was defined by a maximum force criterion of f_{max} = 0.01 eV/Å

DFT Chemical Shift Calculations: For each UMA-optimized structure, NMR shieldings were calculated at the DFT B3LYP level of theory in Gaussian16 (12), employing a three-tier, locally dense basis set (13) with the high-layer atoms (defined above) at 6-311+G(2d,p), atoms within 4 Å of the high layer at 6-311G(d,p), and all remaining atoms at 6-31G. Shieldings were converted to chemical shifts using the linear rescaling relationships derived from the benchmark studies below:

^{13}C: $$\delta_{calc}[DSS(l)] = 178.96 - 0.9813\,\sigma_{calc} \qquad (S1)$$

^{15}N: $$\delta_{calc}[NH_3(l)] = 230.73 - 0.9853\,\sigma_{calc} \qquad (S2)$$

^{1}H: $$\delta_{calc}[TMS(l, neat, MAS)] = 30.77 - 0.9841\,\sigma_{calc} \qquad (S3)$$

Benchmarking Against Solid-State NMR Chemical Shift Test Sets: The full UMA+DFT workflow was benchmarked against our previously established solid-state NMR test sets (14-16). The

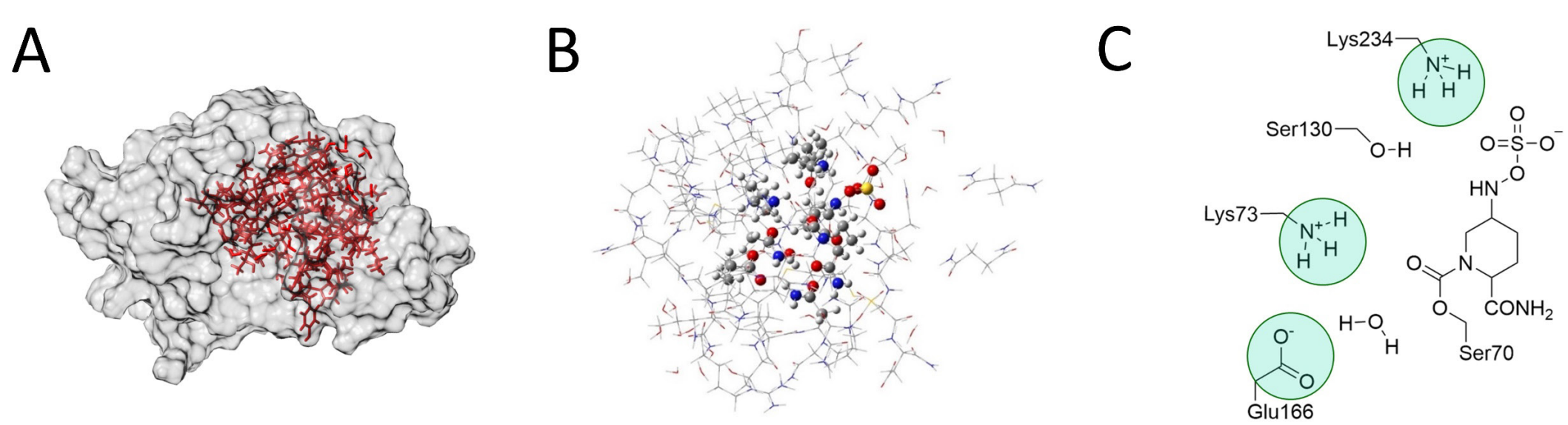

Fig. S1: **Active site cluster construction:** (A) X-ray framework of the Toho-1:avibactam complex (PDB ID 9ZQQ) with the active site highlighted in red; (B) DFT cluster model used for UMA geometry optimization and DFT chemical shift calculations with the high-layer shown in ball-and-stick; (C) Detail of the residues Lys234, Lys73, and Glu166, where protonation states were systematically varied.

benchmark data sets contain 121 ^{13}C, 112 ^{15}N, and 80 ^{1}H experimental shifts from 71 crystal structures. These structures include amino acids, DNA bases, sugars, small drugs, and other small organic molecules. In addition to H, C, N, and O, a few of the crystals contain S or Cl. The molecules are mostly uncharged, but some zwitterions and chlorine salts are contained in the sets.

In brief, each test-set structure was geometry optimized with unit cell dimensions fixed at their experimental values using the ASE/LBFGS/UMA protocol described above. NMR shieldings were then computed using a cluster/fragment protocol in which the shieldings of the central monomer were evaluated explicitly including all dimer interactions with neighboring monomers within 4 Å, while treating more distant environment effects implicitly using PCM. A locally dense basis-set strategy was employed, with 6-311+G(2d,p) on the central monomer, 6-311G(d,p) on neighboring atoms within 2 Å, and 6-31G on all remaining atoms.

Linear rescaling parameters were obtained by least-squares fitting of the experimental chemical shifts, δ_{exp}, versus the computed shielding, σ_{calc}; the resulting fits and residuals are shown in Fig. S2, and the corresponding linear rescaling relationships are given above (Eqs. S1-S3). The REMSEs were calculated from the residuals, $\delta_{exp}-\delta_{calc}$, yielding 1.1 ppm for ^{13}C, 4.0 ppm for ^{15}N, and 0.59 ppm for ^{1}H, identical to earlier all-QM approaches (15, 17). These were subsequently used in defining the weights in the reduced-χ^2 rankings. Corresponding RMSEs for chemical shift tensor components were set at 4.2 ppm for ^{13}C and 8.0 ppm for ^{15}N, in keeping with previous studies (18).

Statistical Methods:

Reduced-χ^2 Analysis: The structural models were ranked using the reduced-χ^2 statistic, a quantitative measure of agreement between first-principles predicted chemical shifts and the experimental NMR parameters, defined as (19, 20)

$$\chi_r^2(\text{model}) = \frac{1}{N}\sum_i \frac{\left(\delta_i^{\text{model}} - \delta_i^{\text{exp}}\right)^2}{s_i^2} \quad \text{(S4)}$$

In this expression, the sum runs over the *N* measured NMR shifts in the high layer (isotropic and tensor principal components); only sites whose local geometry is unconstrained – i.e., whose relevant bonded neighbors are also free to move – are included in the reduced-χ^2 analysis. δ_i^{exp} is the experimental shift; δ_i^{model} is the corresponding predicted shift for a given model; and $1/s_i^2$ is the nuclide- and observable-specific weight assigned by setting s_i to the benchmark RMSE from the corresponding benchmark study. Importantly, the use of the reduced-χ^2 enables chemical shift data from multiple nuclides (^{13}C, ^{15}N, etc.) and of different types (isotropic shifts, tensor principal components) to be combined into a single statistic. With *a priori* weights, the reduced-χ^2 provides a rigorous test of model consistency with the full experimental dataset (19, 20).

Bayesian Probability Analysis: The overall confidence of each model corresponding to the experimental structure was quantified using the Uniform Chi-Squared (UC) Model, a hierarchical Bayesian approach, as previously described (19, 20). In brief, given a set of candidate structural models, *M*, each with a set of first-principles predicted NMR parameters, d_M, corresponding to experimental observables, d^*, the UC Model assigns the posterior probability, $P_{UC}(M|d^*)$, that a candidate model is the experimental structure. The UC model is based on three assumptions:

1. Closed candidate set: the experimental structure is contained within the candidate set $\{M\}$.
2. Gaussian prediction errors: deviations d_M - d^* are independent, normally distributed, and characterized by known standard deviations. This assumption can be validated by examining the distribution of residuals in benchmarking studies, (19, 20) such as the recent work of Dračínský et al. (17).
3. Uniformity in reduced-χ^2 space: the (implicit) space of plausible candidate structures is uniformly distributed with respect to reduced-χ^2. This assumption agrees well with the empirically observed distribution of models in NMR crystallography (4, 21).

The UC Model provides a more cautious estimate of candidate model probabilities than previous approaches (22), assigning decreased likelihood to the best-fit model and increased likelihood to alternate candidates.

Positional Uncertainties: The positional uncertainties in the NMR-refined crystal structure were estimated following the method of Hofstetter and Emsley (23), adapted for our cluster model approach and incorporation of Bayesian posterior probabilities. The goal is to quantify the distribution of structures that remain consistent with the NMR data. Here, we define consistent to mean structures that have a Bayesian UC Model probability ≥ 5%. We refer to this threshold as the UC Model 5% probability cutoff, UC05. Our protocol follows our previously described procedure (7)

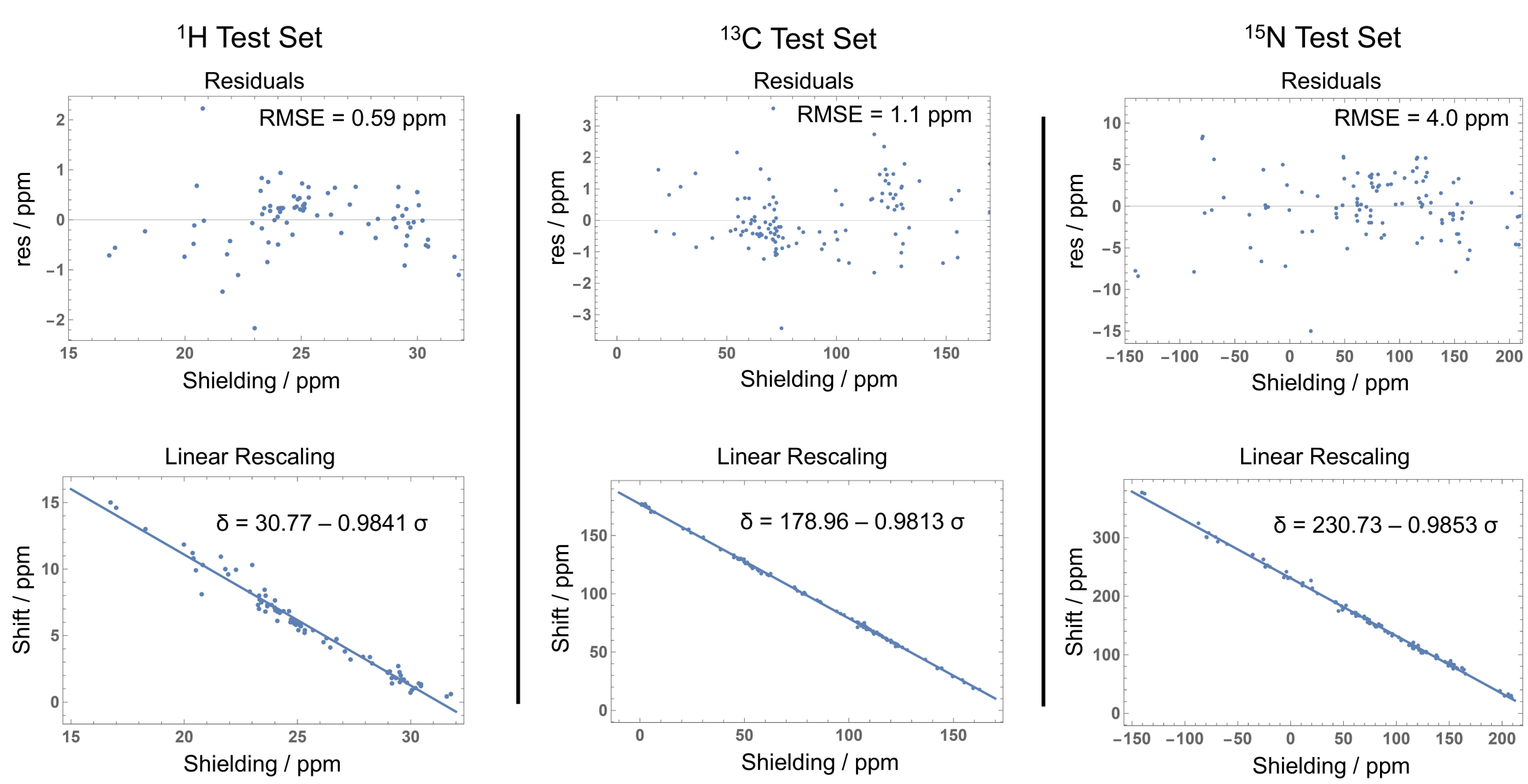

Fig. S2: Linear rescaling of computed NMR shieldings to chemical shifts and corresponding residuals. Bottom panels: experimental chemical shifts (δ) plotted against computed absolute shieldings (σ) for ^{1}H, ^{13}C, and ^{15}N. Top panels: fit residuals after linear rescaling.

with some modifications in the generation of the perturbed structural ensembles, which here are generated by sampling along the normal modes; it consists of the following steps:

1. An ensemble of slightly perturbed structures is generated by stochastic sampling along the normal modes of the active-site cluster

A reduced-size active-site cluster was first generated by selecting all atoms within 3.5 Å of Ser70, Lys73, Glu166, and the covalently bound avibactam; this initial cluster was then expanded and processed as described above. The resulting cluster contained 332 atoms and included residues Cys69, Ser70, Thr71, Ser72, Lys73, Val74, Ala77, Ser130, Asp131, Asn132, Asn135, Thr165, Glu166, Pro167, Leu169, Asn170, Lys234, Thr235, Gly236, Ser237, 5 crystallographic waters, and the covalently bound avibactam molecule. The cluster normal modes were calculated in Gaussian16 at the B3LYP level of theory and 6-31G(d) basis.

An ensemble of (slightly) perturbed structures was next generated by stochastically sampling displacements along the normal modes. For each mode in turn, the displacement amplitude was independently drawn from a normal distribution with a standard deviation set to the classical rms stretch amplitude of that mode

$$x_{rms} = \sqrt{\frac{k_B T}{k}} \quad \text{(S5)}$$

Here, k_B is the Boltzmann constant, T is the temperature (K), and k is the mode force constant (reported in mdyne/Å in the Gaussian output, converted to N/m). Two hundred structures were generated at each temperature from 1 K to 10 K in 1 K increments, and from 10 K to 100 K in 5 K increments, for a total of 5600 structures.

2. First principles chemical shifts and reduced-χ^2 are calculated for the active site region of each structure

For each of the 5600 structures in the ensemble, first principles chemical shifts were calculated at the DFT level of theory (B3LYP/6-31G). These shifts were used to approximate the corresponding chemical shifts for the perturbed structures at the higher level of theory using a Δ correction approach, in which the high-level shift for a perturbed structure was obtained by adding the low-level perturbation to the high-level reference:

$$\delta^{high}\left(\Delta\right) \approx \delta_0^{high} + \left[\delta^{low}\left(\Delta\right) - \delta_0^{low}\right]. \quad \text{(S6)}$$

Here, $\delta_0^{low}(\Delta)$ and $\delta_0^{high}(\Delta)$ are the chemical shifts for a given perturbed structure at the low and (approximated) high levels, and δ_0^{low} and δ_0^{high} are the corresponding shifts for the unperturbed reference structure. Reduced-χ^2 values were then computed for each perturbed structure.

3. Anisotropic displacement parameters are defined at each atom by the correlation of positional deviations with reduced-χ^2 and the UC05 cutoff

The positional deviations of each atom relative to the reference structure were used to construct a covariance matrix of displacements and to define an atom-specific principal axis system (23). For each atom, I, the positional deviations projected onto the PAS axes (r_i) were analyzed jointly with the corresponding reduced-χ^2. Fig S3 shows an example scatter plot of positional variance versus

reduced-χ^2. At fixed reduced-χ^2, the distribution of displacements along each PAS axis is well-approximated by a normal distribution.

For each atom and each principal axis, we define the anisotropic displacement parameter (ADP) as the standard deviation of the displacement distribution evaluated at the bounding surface for structures with UC model probabilities ≥ 5% (UC05). The UC posterior probability depends on both the reduced-χ^2 for the best-fit model and the number of degrees-of-freedom. For the 23 degrees of freedom in the Toho-1:avibactam analysis, the UC05 cutoff corresponds to $\chi^2_{r,\mathrm{UC05}} = 1.8\chi^2_{r,\mathrm{best\ fit}} = 1.75$

To determine the value of the ADPs, structures were binned by reduced-χ^2, and for each bin the standard deviation of the resulting displacement distribution along each PAS axis was computed. The dependence of this standard deviation on reduced-χ^2 was then fit to the functional form

$$\Sigma_{i,l}\left(\chi_r^2\right) = \sigma'_{i,l}\sqrt{\chi_r^2} \qquad \text{(S7)}$$

put forward by Hofstetter and Emsley (23), and evaluated at the reduced-χ^2 value corresponding to UC05. The NMR-derived ADPs were then reported as

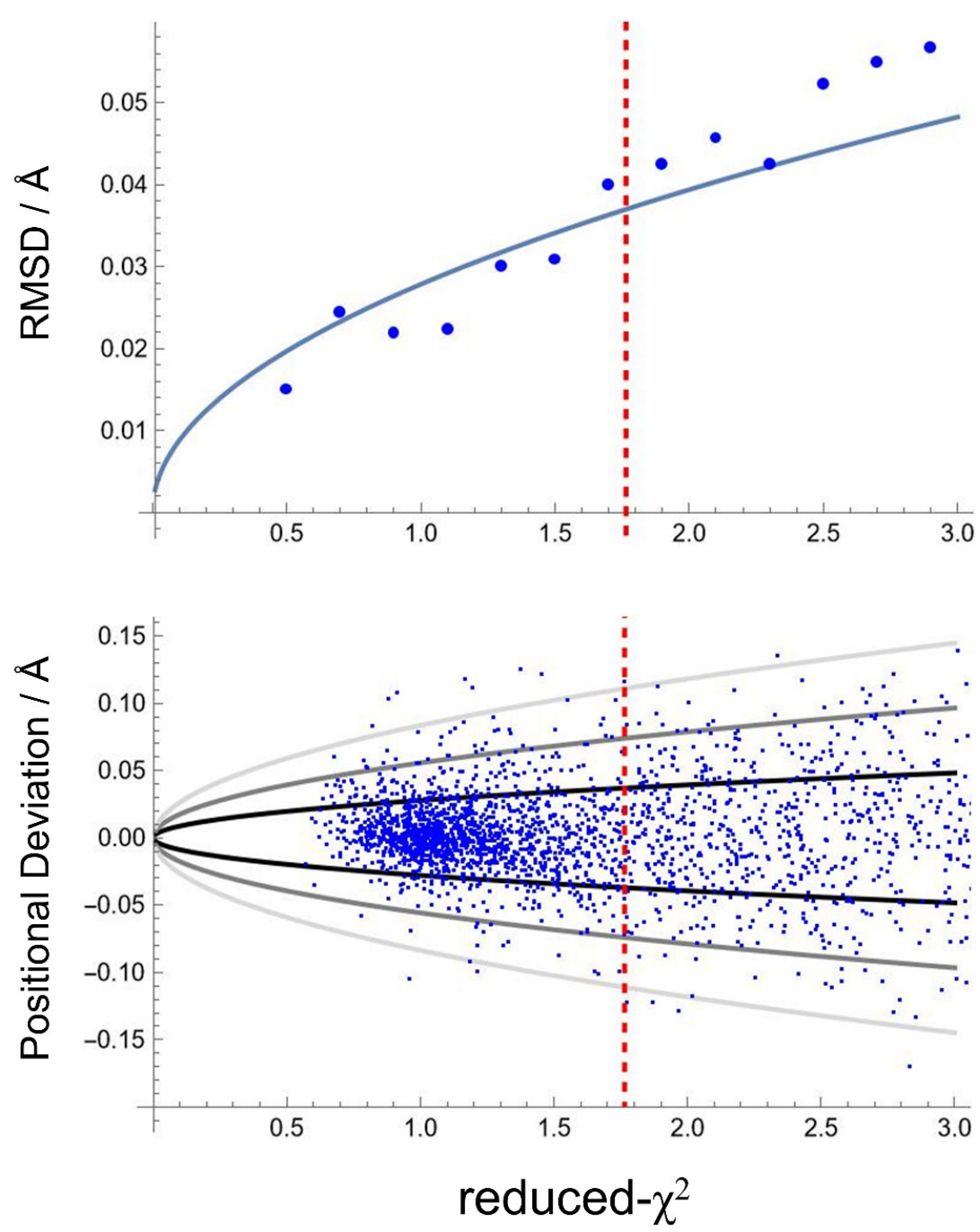

Fig. S3: Positional uncertainties in NMRX. Bottom: Scatter plot of reduced-χ^2 and positional deviation along one principal axis of the anisotropic displacement tensor for C$^{\beta}$ of Ser70. The vertical line at reduced-χ^2 = 1.75 marks the UC05 cutoff. The black and gray lines indicate ±1, 2, and 3 times the standard deviation of the structure distribution at a given reduced-χ^2. Top: Root-mean-square-deviation of the structural distribution as a function of reduced-χ^2, along with the best fit to Eq. S7.

$$U_{ii,l}^{\mathrm{PAS}} = \sigma_{i,l}'^{2} \chi_{r,UC05}^{2} \quad \text{(S8)}$$

The ADPs were transformed back to the coordinate frame of the crystal structure using standard tensor methods (6, 24), and incorporated into an edited version of the protein crystal structure file together with the refined active-site coordinates from NMR crystallography. This file is included as Supplementary Dataset 1: Dataset_1_9ZQQ_active_site_refined_NMRX.pdb.

4. Reported ADPs:

ADPs are reported only for atoms within the high layer of the cluster models. Small displacements of atoms at the periphery of the cluster are expected to have minimal influence on the high-layer chemical shifts used in the reduced-χ^2 analysis and are therefore expected to be weakly constrained by the NMR data. As reported in the main text, there is no significant difference between the ADP for the active-site side chains and those for the covalently bound avibactam, despite the fact that only the amino acid side-chain chemical shifts were included in the reduced-χ^2 analysis. In contrast, ADPs for atoms at the periphery of the cluster are nearly twice as large. This is consistent with the local sensitivity of ^{13}C and ^{15}N chemical shifts to geometry within ~2-4 Å (14), with structural perturbations beyond this range have diminishing impact.

Solid-State NMR Backbone and Side-chain Assignments

Backbone assignments for the Toho-1:avibactam complex were obtained using the protocol established in our previous solid-state NMR study of free Toho-1 (25). Avibactam binding produces only minor backbone chemical shift changes for most residues, enabling direct transfer of assignments; this is illustrated in Fig. S4 which compares the unbound NCA spectrum acquired at 1.1 GHz with the avibactam-bound NCA spectrum collected at 900 MHz. Residues with larger perturbations were assigned using backbone-walk connectivities from 3D CANCO, 3D NCACX, and 3D NCOCX; an example backbone walk is shown in Fig. S5.

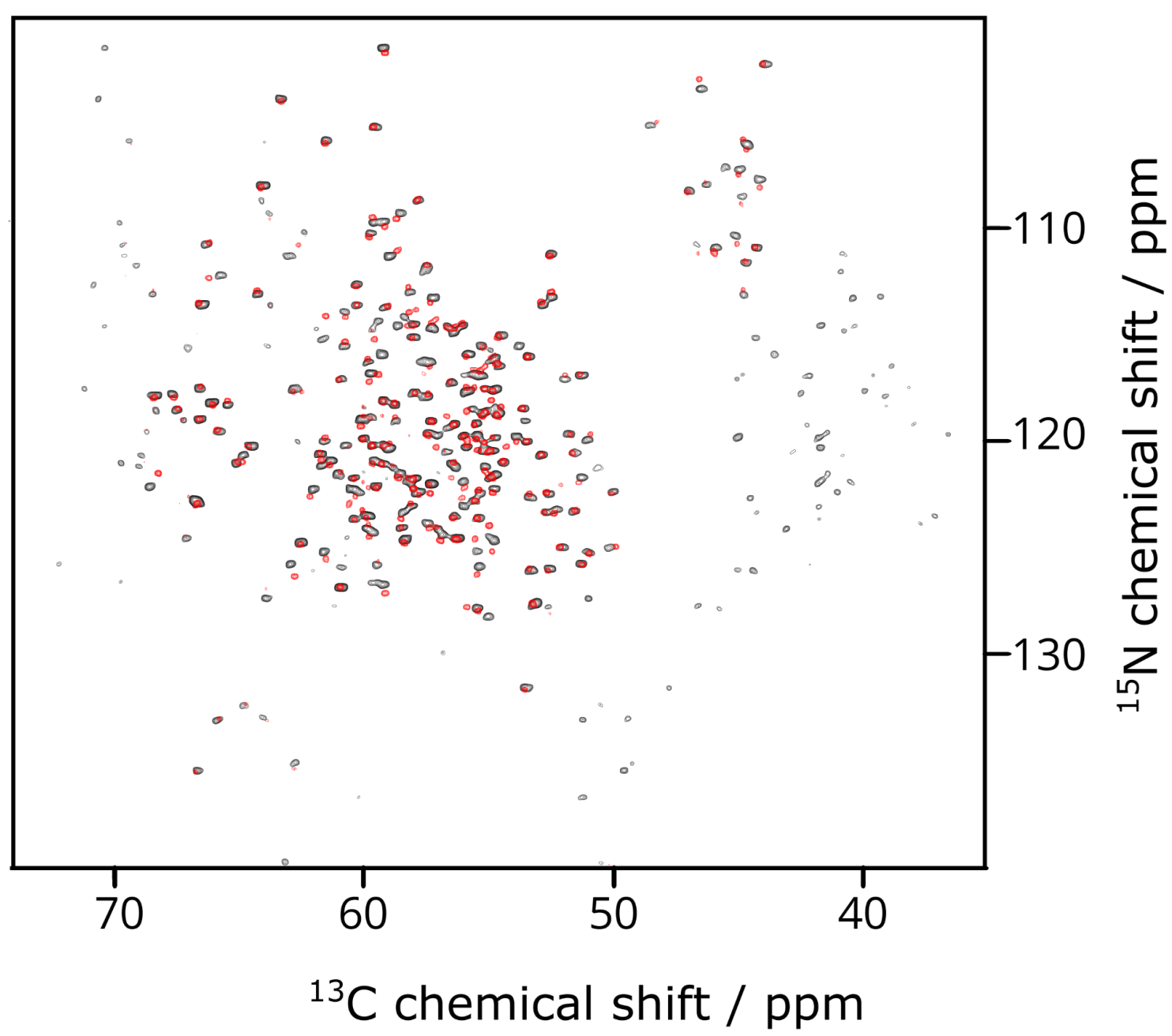

Fig. S4: Comparison of the 2D NCA for U-^{13}C,^{15}N-Toho-1 in the presence (black) and absence (red) of avibactam acquired at 900 MHz and 1.1 GHz, respectively, and incorporating LOW-BASHD ^{13}C homonuclear decoupling during detection (3). The spectrum demonstrates the high resolution achievable for this microcrystalline protein sample. Data acquired at 40 kHz MAS and 273 K.

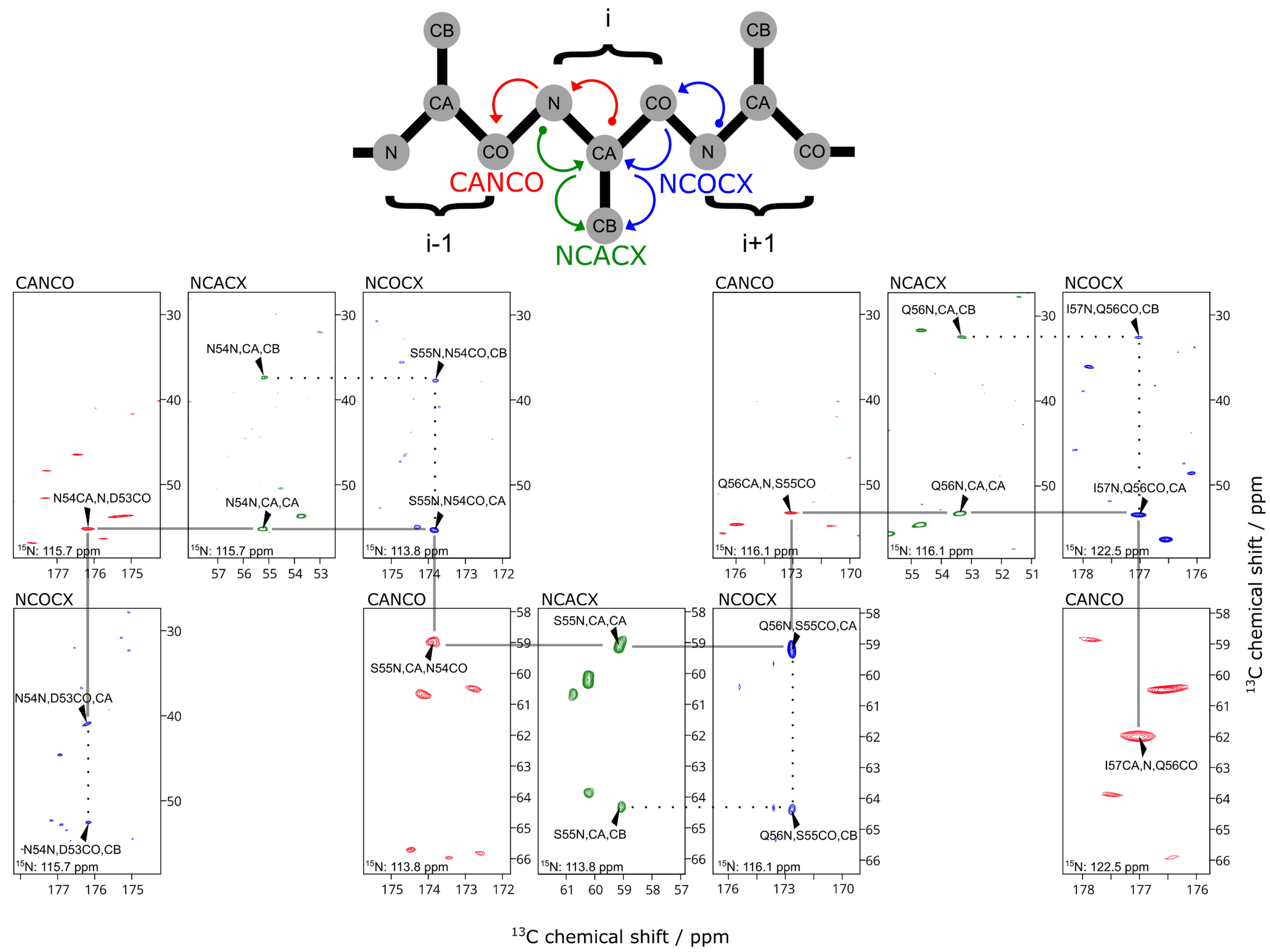

Fig. S5: Two-dimensional strip plots illustrating the backbone walk from N54 to I57 using 3D NCACX (green), CANCO (red), and NCOCX (blue) spectra. Solid lines trace the backbone connectivity, while the dashed lines add confirmation through the C^{β} side-chain carbons. The amide nitrogen chemical shift for each plane is labeled in the bottom corner of the corresponding strip. Data acquired at 900 MHz (^{1}H), 40 kHz MAS, and 273 K.

Side-chain assignments were obtained from a complementary set of 2D and 3D ^{13}C-^{13}C and ^{15}N-^{13}C correlation spectra. At 900 MHz, we acquired 2D NCA, 3D NCACX, 3D CANCO, and 3D NCOCX spectra, together with a 2D 50 ms ^{13}C-^{13}C DARR spectrum; a 3D CCC spectrum was also collected at 600 MHz. Side-chain carbon assignments followed a stepwise workflow. Initial assignments were established from the 3D NCACX spectrum (50 ms DARR mixing). These assignments were then validated and extended using the 2D 50 ms CC-DARR and 3D CCC experiments. Final confirmation and completion were achieved with an NCACX spectrum (200 ms DARR mixing) acquired with a high-sensitivity 3.2 mm MAS cryoprobe at 600 MHz, which provided near-complete carbon-carbon correlation coverage with extensive connectivity to adjacent residues, enabling detailed side-chain characterization and sequential correlations extending to ±2 residues (Fig. S6).

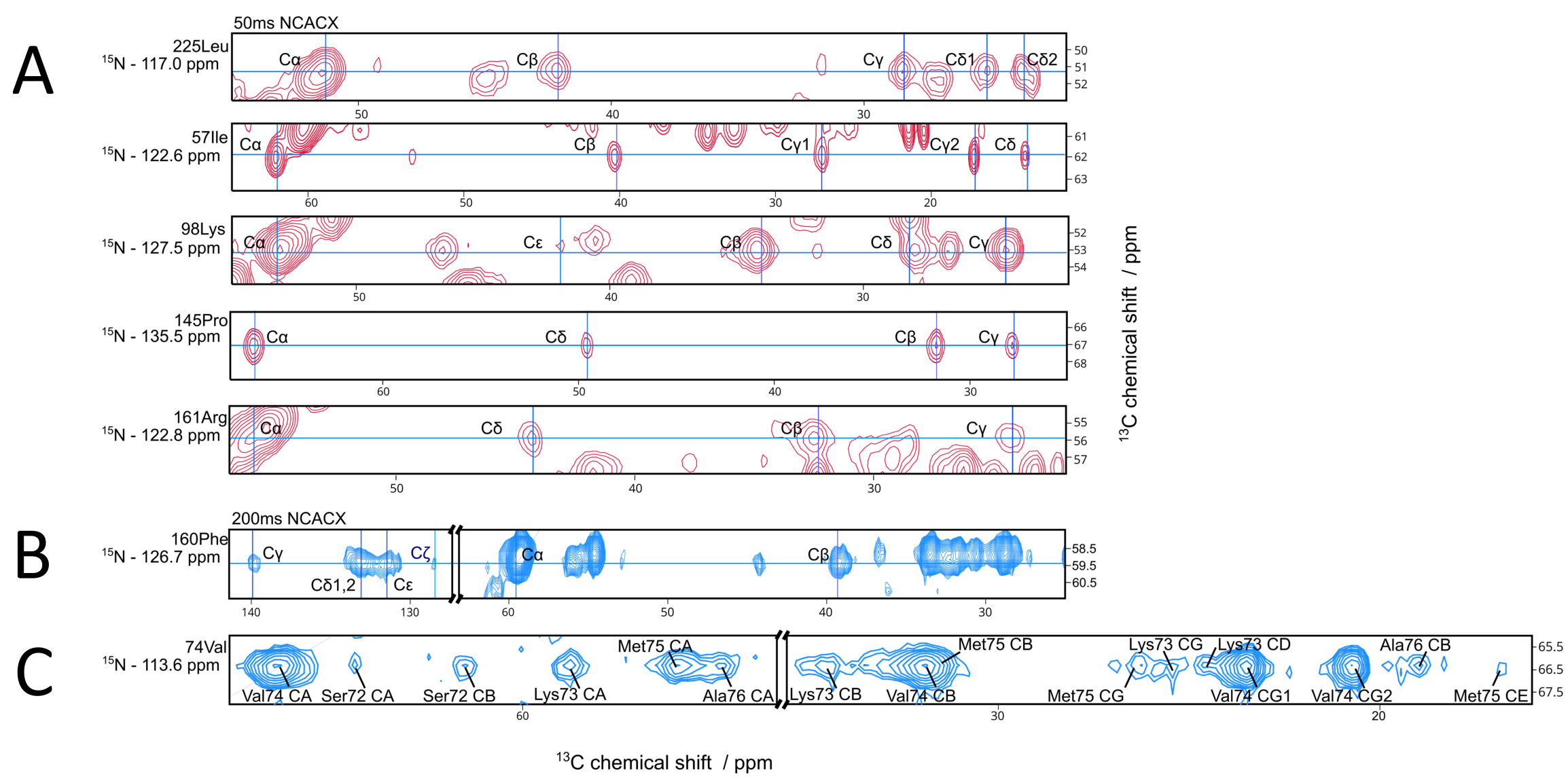

Fig. S6. NCACX strip plots illustrating side-chain assignment completeness. (A) 3D NCACX (50 ms DARR mixing) showing extensive aliphatic side-chain correlations for representative residues Leu225, Ile57, Lys98, Pro145, and Arg161. (B) 3D NCACX (200 ms DARR mixing) for Phe160, extending correlations across the full aromatic side chain. (C) 3D NCACX (200 ms DARR mixing) for Val74, showing additional long-range correlations to neighboring residues Ser72, Lys73, Met75, and Ala76. Data acquired at 600 MHz on a CPMAS CryoProbe at 13.5 kHz and 268 K.

Side-chain nitrogen resonances were assigned using a combination of the above experiments and several side-chain specific experiments. For example, when acquired with sufficient spectral width, the NCA experiment at 900 MHz allowed the assignment of C^{δ}-N^{ε} correlations for arginine residues. Additional assignments were obtained using an NcaCX 2D correlation experiment on the MAS cryoprobe at 600 MHz that links C^{ζ} to both N^{ε} and N^{η} nuclei, providing unambiguous identification of distal arginine nitrogen sites. This same experiment allowed for the assignment of tryptophan $N^{\varepsilon 1}$. Nearly all asparagine (14 of 15) and all glutamine (12 of 12) side-chain amino groups were assigned via the NCACX and NCOCX 3D experiments at 900 MHz. Lys N^{ζ} were assigned using a combination of 2D NZCX and 3D NCANZ 3D experiments (Figs. 3 and S7).

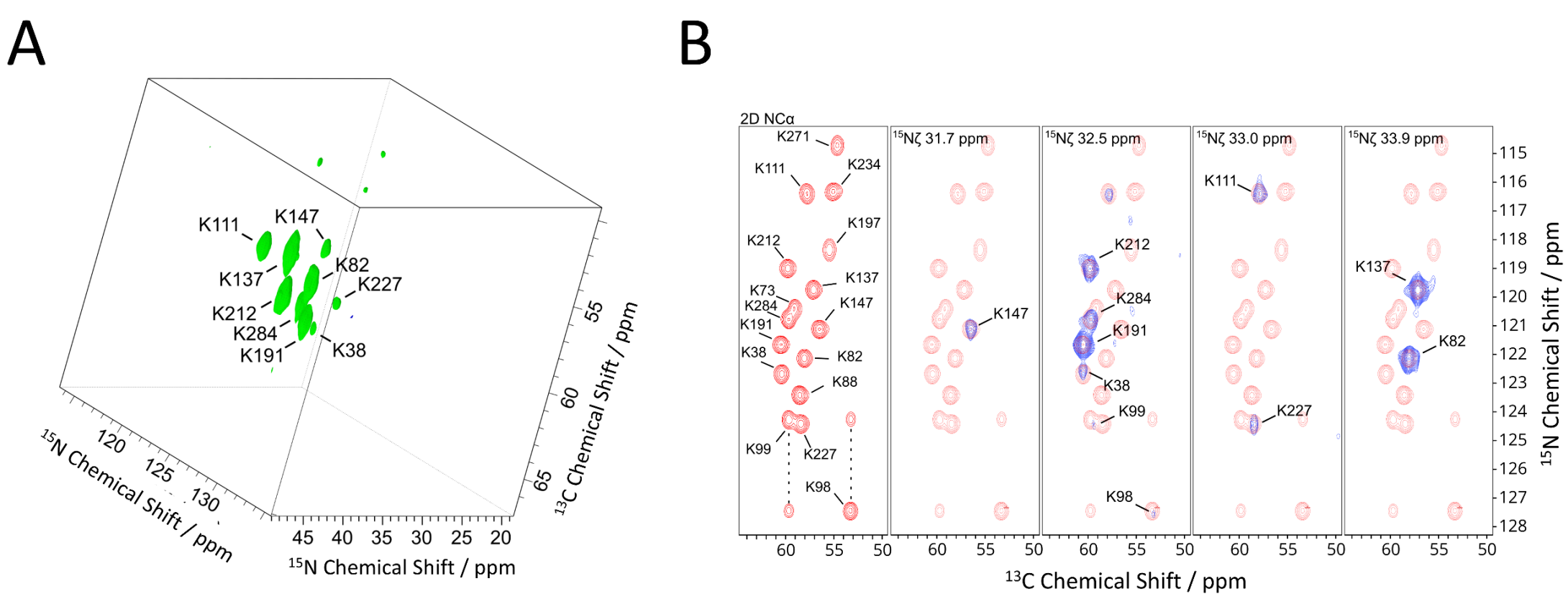

Fig. S7: Lys N^{ζ} assignments. (A) 3D NCANZ used to connect the assigned backbone resonances to lysine side-chain N^{ζ} sites. (B) Matching of 2D planes to the NCA spectrum enables assignment of the 14 remaining Lys N^{ζ} resonances. Data acquired at 600 MHz on a CPMAS CryoProbe at 13.5 kHz MAS and 268 K.

Assignment of the pH-Dependent Lysine Resonance to Lys137

Previous studies of the CTX-M-9:avibactam complex in solution identified an ^{1}H-^{13}C HSQC cross peak at C^{ε}~ 42.5 ppm and H^{ε}~ 2.65 ppm that exhibited pH-dependent chemical-shift perturbations as the pH was decreased to 4.5. This resonance was tentatively assigned to Lys73 and interpreted as evidence of an unusually low pK_a (< 6.5) for the active-site residue (26).

We observe an analogous isolated cross peak in the solution-state ^{1}H-^{13}C HSQC of U-^{13}C,^{15}N-Lys Toho-1:avibactam (Fig. S8). This resonance has nearly identical chemical shifts and pH-dependent behavior to the peak reported for CTX-M-9:avibactam (Fig. S8C), suggesting that the two resonances arise from the same conserved lysine environment

To assign this resonance in the Toho-1:avibactam complex, we used a long-range solution-state CH-TOCSY experiment to correlate the pH dependent H^{ε} resonance to its full side-chain ^{13}C chemical shifts. These solution-state NMR data were then compared to the corresponding 2D planes from the solid-state NCACX spectrum (Fig. S8B), allowing definitive assignment of the pH-dependent spin system to Lys137. In contrast, the Lys73 side-chain resonances (shown in faint gray in Fig. S8B) do not overlap with those for this pH-dependent peak.

Lys137 is a surface-exposed residue (Fig. S8A); its pH-dependent chemical shift behavior is consistent with a standard lysine side chain experiencing a local environmental change, rather than a perturbed active-site pK_a.

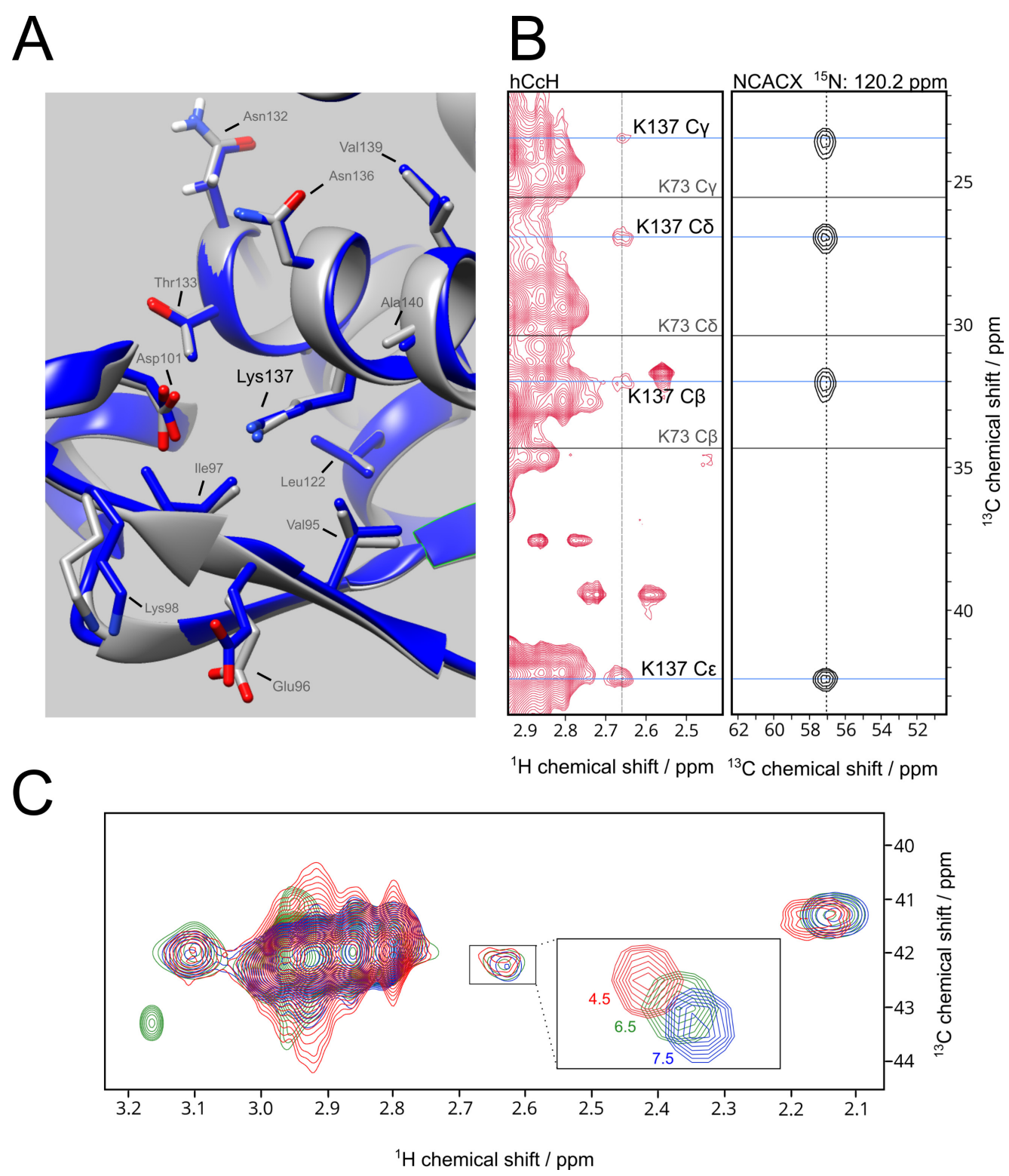

Fig. S8: Assignment of the pH-dependent lysine resonance to Lys137. (A) Superposition of the crystal structures for the Toho-1:avibactam complex (PDB ID 9ZQQ) in gray and the CTX-M-14:avibactam complex (PDB ID 6MZ2) in blue. There is no crystal structure for CTX-M-9 in complex with avibactam, but its sequence differs from CTX-M-14 by a single A231V substitution. (B) Comparison of the solution-state long-range CH TOCSY spectrum of U-^{13}C,^{15}N-Lys labeled Toho-1:avibactam (red, left) and the corresponding 2D plane of the solid-state NCACX spectrum (black, right), allowing assignment of the pH-dependent lysine spin system to Lys137. For comparison, the Lys73 side-chain resonances are shown as the faint gray lines. (C) pH dependence of the Lys137 C$^{\varepsilon}$-H$^{\varepsilon}$ resonance in the solution state ^{1}H-^{13}C HSQC spectra at pH: 4.5 (red), 6.5 (green), and 7.5 (blue). Solution-state data acquired at 700 MHz on a Bruker Avance III spectrometer equipped with a TCI cryoprobe.

Expanded Materials and Methods

Protein Preparation and Crystallization

Uniform-^{13}C,^{15}N Toho-1 was expressed, purified, and crystallized as previously described (25). Upon crystallization, avibactam was allowed to soak in by adding it directly to the crystals in crystal buffer at 7mM for 30 minutes. For data collection on the 600MHz MAS CryoProbe, the sample was packed in a Bruker 3.2mm CryoProbe rotor. For data collection at 900MHz and 1.1GHz, samples were packed in a 1.6mm Varian style rotor SSNMR.

X-ray Crystallography

1.1 Å Resolution Structure: The Toho1 β-lactamase enzyme was over-expressed using an E. coli based expression system and purified as previously described (27-30). Crystals were grown at 20 °C using 30 µl of 10 mg/ml protein concentration in a solution containing 2.0 M ammonium sulfate and 0.2 M sodium citrate (pH 6.1) using the batch method. For soaking, crystals were placed in a solution containing 2.7 M ammonium sulfate, 0.1 M sodium citrate (pH 6.1), and 200 mM Avibactam for 24 hours. Crystals used in X-ray experiments were momentarily placed in a reservoir solution containing a cryoprotectant (30% w/v trehalose) and subsequently flash-frozen in liquid nitrogen. X-ray data was collected at 100 K on the IMCA-CAT beamline at the Advanced Photon Source, reduced using XDS (31), and then scaled with Scala from the CCP4 suite9 (32). The PHENIX suite (33) was then used to refine the data to convergence with model building using the Coot molecular graphics program (34). The data collection and refinement statistics are summarized in Table S1.

2.0 Å Resolution Structure: Crystals for the 2.0 Å structure were grown at 4 °C using 500 µl of 10 mg/ml protein concentration in a solution containing 6.5% PEG-8k, 7.5 mM spermine, and 20 mM MES (pH 6.5) using the batch method. For soaking, crystals were placed in a solution containing an additional 200 mM Avibactam for 24 hours. Crystals used in X-ray experiments were momentarily placed in a reservoir solution containing a cryoprotectant (30% w/v trehalose) and subsequently flash-frozen in liquid nitrogen. X-ray data was collected at 100 K on a Bruker D8 Venture and processed as above. The data collection and refinement statistics are summarized in Table S1.

Table S1: Statistics for X-ray crystallographic data collection and refinement

**PDB**<br>ligand	9ZO2<br>Avibactam	9ZOZ<br>none	9ZQQ<br>Avibactam
**Data collection**			
Detector	Dectris PILATUS 6M detector	Photon III	Photon III
Space group	P 32 2 1	P 21 21 21	P 21 21 21
Unit cell, a, b, c (Å)	72.67, 72.67, 97.93	44.13, 73.35, 74.83	44.83, 73.30, 78.39
Unit cell, α, β, γ (°)	90.00, 90.00, 120.00	90.00, 90.00, 90.00	90.00, 90.00, 90.00
Wavelength (Å)	1.0	1.54, Cu Kα	1.54, Cu Kα
Wilson B Value ($Å^2$)	10.2	15.9	22.2
Resolution (Å)	38.65 - 1.15<br>(1.21 -1.15)	22.06 – 2.00<br>(2.05-2.00)	23.33 - 2.00<br>(2.05-2.00)
R_{sym}	0.047 (0.396)	0.1379 (0.5637*)	0.0925 (0.4714*)
R_{meas}	0.052 (0.442)	0.191(0.876)	0.212 (1.145)
R_{pim}	0.021 (0.193)	0.078(0.389)	0.050 (0.327)
I / σI	17.6 (3.70)	8.5 (2.3)	10.1 (2.5)
CC1/2	0.99 (0.89)	0.992 (0.720)	0.998 (0.851)
Completeness (%)	97.70 (99.80)	99.9 (100)	99.9 (99.9)
Multiplicity	5.5 (5.0)	5.9 (5.0)	17.4 (12.1)
**Refinement**			
Resolution (Å)	36.34 – 1.15	22.06 – 2.00	23.33 - 2.00
Reflections (R_{Free})	104185 (5022)	100773 (6212)	315438(16081)
R_{work} / R_{free} [a,b] (%)	13.35 / 15.12	18.3 / 23.8	19.9 / 25.4
Number of Atoms (Protein, Ligand, SO4, H2O)	4410<br>(4017, 55, 10, 328)	2262<br>(1964, 0, 0, 298)	2217<br>(1969, 34, 0, 214)
Average B factor ($Å^2$) (Protein, Ligand, Water)	14.82<br>(12.81, 13.10, 27.51)	17.30<br>(16.31, NA, 23.83)	24.07<br>(23.47, 25.77, 29.34)
R.M.S. Bond lengths (Å)	0.007	0.28	0.30
R.M.S. Bond angles (°)	1.098	0.47	0.47
Ramachandran plot (%)			
Most Favorable	98.00	98	98
Allowed	2.0	2	2
Outliers	0	0	0

Values in parentheses are for highest-resolution shell.
[a] *R*work = Σhkl |Fo(hkl) − Fc(hkl)|/Σhkl Fo(hkl).
[b] *R*free was calculated for a test set of reflections (5%) omitted from the refinement.
*Refers to shell 2.10-2.00 Å, calculated with Xprep.

NMR Spectroscopy

Solid-State NMR**:**

Solid-state NMR spectra were collected at NMRFAM (900 MHz) and UC Riverside (600 MHz, MAS CryoProbe). All ^{13}C spectra were referenced to DSS using adamantane as a secondary external standard (35, 36); ^{1}H and ^{15}N chemical shifts were referenced indirectly to DSS and liquid-NH_3 (25 °C), respectively, via the frequency ratio method (35). Hardware employed and environmental conditions are summarized in Table S2.

Table S2: Hardware and Environmental Conditions

Spectrometer	Location	Console	Probe	MAS Frequency	VT Set Point
900	NMRFAM	AVANCE III	1.6 mm Black Fox HCN	20 KHz	0 °C
900 MHz (CCC)	NMRFAM	AVANCE NEO	2.5 mm Black Fox HCN	20.833 kHz	-5 °C
600 MHz	UCR	AVANCE NEO	3.2 mm HCN MAS CryoProbe	13.5 kHz	-5 °C

Assignment Suite at 900 MHz**:**

All experiments employed adiabatic CP with a downward tangential ramp on ^{1}H for initial polarization (37), SPECIFIC CP with an upward tangential ramp for C→N and N→C transfers (38), DARR mixing with a ^{1}H RF amplitude of 20 kHz for C→C transfers (39), and SPINAL-64 (100 kHz) for ^{1}H decoupling during acquisition (40). When appropriate, a 4.7 μs ^{13}C π-pulse was applied at the center of indirect evolution periods on nitrogen to decouple $^{1}J_{NC}$; during the second indirect evolution period of NCACX and NCOCX experiments, a 4.7 μs ^{13}C hard π-pulse, a 270 μs ^{13}C soft π-pulse with an RSNOB shape at 175 (NCACX) or 55 (NCOCX) ppm, and a 9.2 μs ^{15}N π-pulse were applied to decouple $^{1}J_{C\alpha CX}$ and $^{1}J_{NC}$. (41). A 1 s relaxation delay was used for all experiments except the CCC, which used 1.5 s. The 3D spectra were acquired using a 25% NUS schedule. Experiment specific parameters are detailed in Table S3.

Table S3: 900 MHz Pulse Sequence Parameters

Experiment	Carrier	CP Contact	Mixing/Transfer	complex points x digitization rate	t_{acq}	total exp time
CC DARR	100 ppm	**HC =** 2.5 ms<br>84 kHz ^{1}H<br>65 kHz ^{13}C	CC DARR 50 ms	1536 x 12.5 μs (t_1)<br>3072 x 10 μs (t_2)	19.2 ms (t_1)<br>30.7 ms (t_2)	15.1 h
NCA	55 ppm ^{13}C<br>120 ppm ^{15}N	**HN =** 1.5 ms<br>49 kHz ^{1}H<br>30 kHz ^{15}N	**NCA=** 8 ms SPCP<br>11 kHz ^{13}C<br>30 kHz ^{15}N	512 x 50 μs (t_1)<br>3072 x 10 μs (t_2)	25.6 ms (t_1)<br>30.7 ms (t_2)	18.4 h
NCACX	55 ppm ^{13}C<br>120 ppm ^{15}N	HN	NCA<br>CC DARR 50 ms	128 x 150 μs (t_1)<br>128 x 100 μs (t_2)<br>3072 x 10 μs (t_3)	19.2 ms (t_1)<br>12.8 ms (t_2)<br>30.7 ms (t_3)	61 h
NCOCX	175 ppm ^{13}C<br>120 ppm ^{15}N	HN	**NCO =** 7.5 ms<br>12 kHz ^{13}C<br>30 kHz ^{15}N<br>CC DARR 50 ms	128 x 150 μs (t_1)<br>100 x 150 μs (t_2)<br>3072 x 10 μs (t_3)	19.2 ms (t_1)<br>15.0 ms (t_2)<br>30.7 ms (t_3)	79 h
CANCO	175 ppm ^{13}C<br>120 ppm ^{15}N	**HCA =** 1.2 ms<br>84 kHz ^{1}H<br>65 kHz ^{13}C	**CAN =** 8 ms<br>12 kHz ^{13}C<br>30 kHz ^{15}N<br>NCO	128 x 100 μs (t_1)<br>128 x 150 μs (t_2)<br>3072 x 10 μs (t_3)	12.8 ms (t_1)<br>18.2 ms (t_2)<br>30.7 ms (t_3)	92 h
CCC	175 ppm ^{13}C	HC	CC DARR 50 ms<br>CC DARR 250 ms	360 x 16 μs (t_1)<br>360 x 16 μs (t_2)<br>3072 x 10 μs (t_3)	5.8 ms (t_1)<br>5.8 ms (t_2)<br>30.7 ms (t_3)	302 h

MAS CryoProbe Experiments at 600 MHz

All experiments employed adiabatic CP with an upward linear ramp (90%-100%) on ^{1}H for initial polarization (37), SPECIFIC CP with an upward linear ramp (80%-100% amplitude on nitrogen) for C→N and N→C transfers (38), DARR mixing with a ^{1}H RF amplitude of 13.5 kHz for C→C transfers (39), and SPINAL-64 (83 kHz) for ^{1}H decoupling during acquisition (40). When appropriate, a 10 μs ^{13}C π-pulse or 16 μs ^{15}N π-pulse was applied at the center of indirect evolution periods to decouple J_{NC}. A 3 s relaxation delay was used for all experiments. Experiment specific parameters are detailed in Table S4.

2D ^{15}N xCSA and 3D ^{13}C DARR-xCSA experiments were performed using the CSA amplification experiment of Zhang and Gan (1, 2), modified for modulo detection (42). The amplifications factors were 36 for ^{15}N and 6 for ^{13}C. For the 3D ^{13}C experiment, a selective, 1 ms Gaussian pulse immediately after CP was used to select a narrow region about 32 ppm (Cγ for Glu166) in the indirect carbon dimension. The MAS rate was 13.589 kHz for both.

Table S4: 600 MHz Pulse Sequence Parameters

Experiment	Carrier	CP Contact	Mixing/Transfer	complex points x digitization rate	t_{acq}	total exp time
NcaCX	55 ppm ^{13}C<br>119 ppm ^{15}N	**HN** = 2 ms<br>52 kHz ^{1}H<br>39 kHz ^{15}N	**NCA**= 4 ms SPCP<br>4.3 kHz ^{13}C<br>10.1 kHz ^{15}N<br>CC DARR 200 ms	256 x 148.15 μs (t_1)<br>2048 x 16 μs (t_2)	37.9 ms (t_1)<br>32.8 ms (t_2)	9.8 h
NCACX	53 ppm ^{13}C<br>122 ppm ^{15}N	HN	NCA<br>CC DARR 50 ms	64 x 740 μs (t_1)<br>32 x 298.5 μs (t_2)<br>2048 x 20 μs (t_3)	47.4 ms (t_1)<br>9.6 ms (t_2)<br>41.0 ms (t_3)	14.8 h
NZCX	40 ppm ^{13}C<br>35 ppm ^{15}N	HN	**NZCE**=4ms SPCP<br>4.3 kHz ^{13}C<br>10.1 kHz ^{15}N<br>CC DARR 200 ms	128 x 148.15 μs (t_1)<br>2048 x 16 μs (t_2)	18.9 ms (t_1)<br>32.8 ms (t_2)	19.5 h
NCANZ	56 ppm ^{13}C<br>125 ppm ^{15}N	HN	NCA<br>CC DARR 200 ms<br>**CENZ** = 5.5 ms<br>9.6 kHz ^{13}C<br>14.3 kHz ^{15}N	32 x 740.74 μs (t_1)<br>32 x 370.38 μs (t_2)<br>1536 x 40 μs (t_3)	23.7 ms (t_1)<br>11.9 ms (t_2)<br>61.4 ms (t_3)	79.5 h
^{15}N EXSY	30 ppm ^{15}N	HN	1-200 ms	128 x 296.3 μs (t_1)<br>4096 x 20 μs (t_2)	26.4 ms (t_1)<br>81.9 ms (t_2)	20.8 h
^{1}H-^{15}N FLSG HETCOR	4 ppm ^{1}H<br>38 ppm ^{15}N	HN	FSLG:<br>57.735 kHz offset<br>81.649 kHz ^{1}H rf	128 x 170 μs (t_1)<br>1024 x 40 μs (t_2)	21.7 ms (t_1)<br>40.9 ms (t_2)	14 h
^{15}N xCSA 2D	45 ppm ^{15}N	HN		64 x 9.2 μs (t_1)<br>2048 x 50 μs (t_2)	588.8 μs (t_1)<br>102.4 ms (t_2)	10.5 h
^{13}C DARR-xCSA 3D	180 ppm ^{13}C<br>32 ppm ^{13}C	**HC =** 2 ms<br>65 kHz ^{1}H<br>51 kHz ^{13}C	CC DARR 5 ms	32 x 592.58 μs (t_1)<br>64 x 4.6 μs (t_2)<br>1536 x 20 μs (t_3)	19.0 ms (t_1)<br>294 μs (t_2)<br>30.7 ms (t_3)	115.5 h

Solution-State NMR Spectroscopy

Solution-state NMR spectra were acquired at 700 MHz on a Bruker Avance III spectrometer equipped with a TCI cryoprobe. The sample consisted of 700 μM U-^{13}C,^{15}N-Lys labeled Toho-1 + 2.8 mM Avibactam in 20 mM MES buffer at pH 6.5. Experiments were performed using standard Bruker library pulse programs for HC HSQC (43-45) (hsqcetgpsisp2.2) and hCcH-TOCSY (46) (hcchdigp2d2), with the latter modified for 2D acquisition by collapsing the 3D experiment's t_2 dimension. Experiment specific parameters are detailed in Table S5.

Table S5: 700 MHz Pulse Sequence Parameters

Experiment	Carrier	Mixing/Transfer	complex points x digitization rate	t_{acq}	total exp time
HC HSQC	100 ppm ^{13}C		2048 x 22.7 μs (t_1)<br>1024 x 110 μs (t_2)	46.5 ms (t_1)<br>112.6 ms (t_2)	4.5 h
hCcH TOCSY	43 ppm ^{13}C	DIPSI III:<br>21.73 ms	256 x 71 μs (t_1)<br>2048 x 102 μs (t_2)	18.2 ms (t_1)<br>208.9 ms (t_2)	36.5 h

Additional Figures

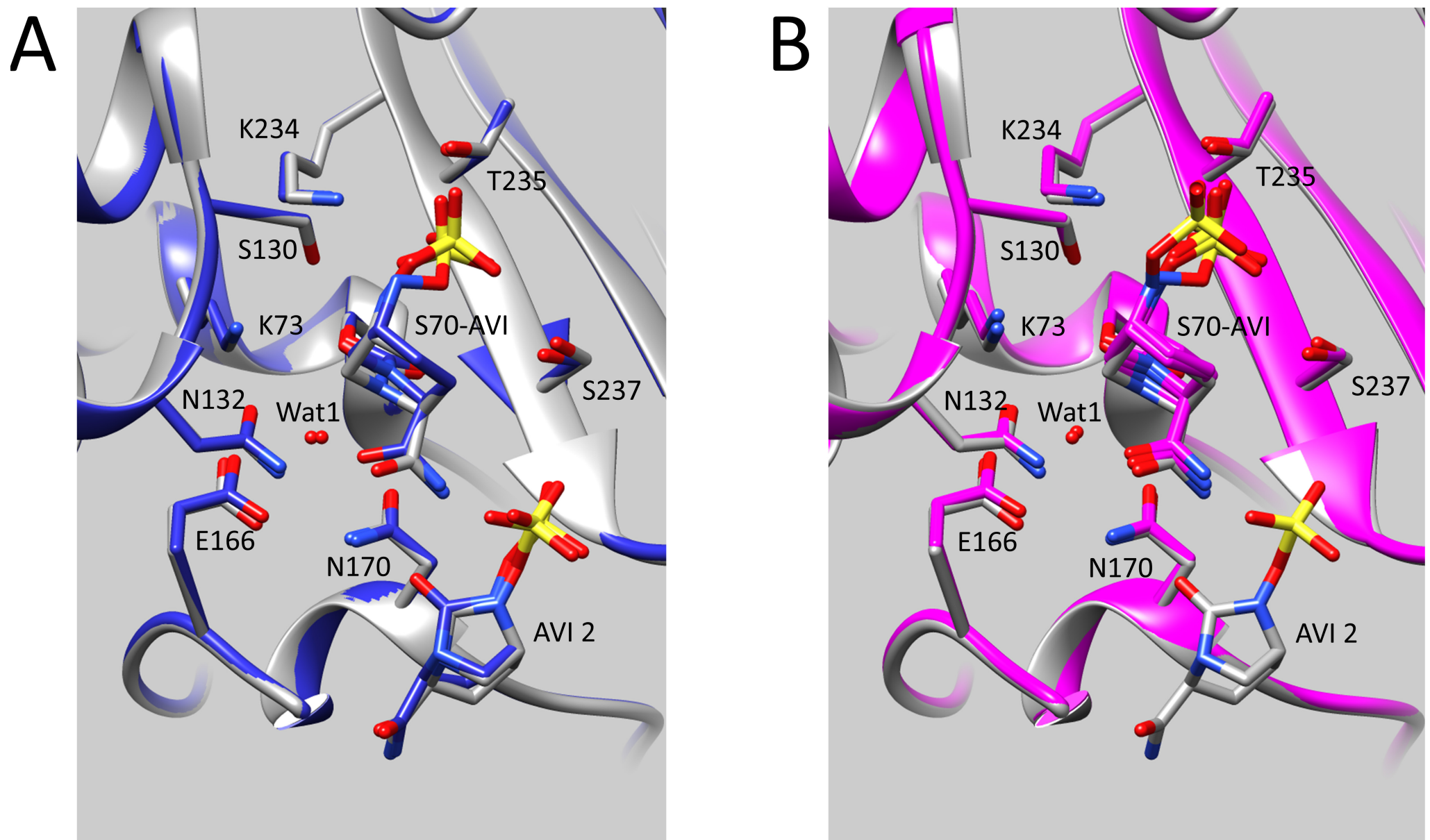

Fig. S9: (A) Superposition of the two Toho-1:avibactam structures: the 1.15 Å, high-salt structure 9ZO2 (blue) and the 2.0 Å, low-salt structure 9ZQQ (gray). The protein backbone and catalytic core are nearly superimposable, with the exception of the conformationally flexible avibactam O-sulfonate group. (B) Overlay of Toho-1:avibactam 9ZQQ (gray) and the pH 7.9 CTX-M-14:avibactam complex 6MZ2 (magenta), highlighting the conserved active-site structure.

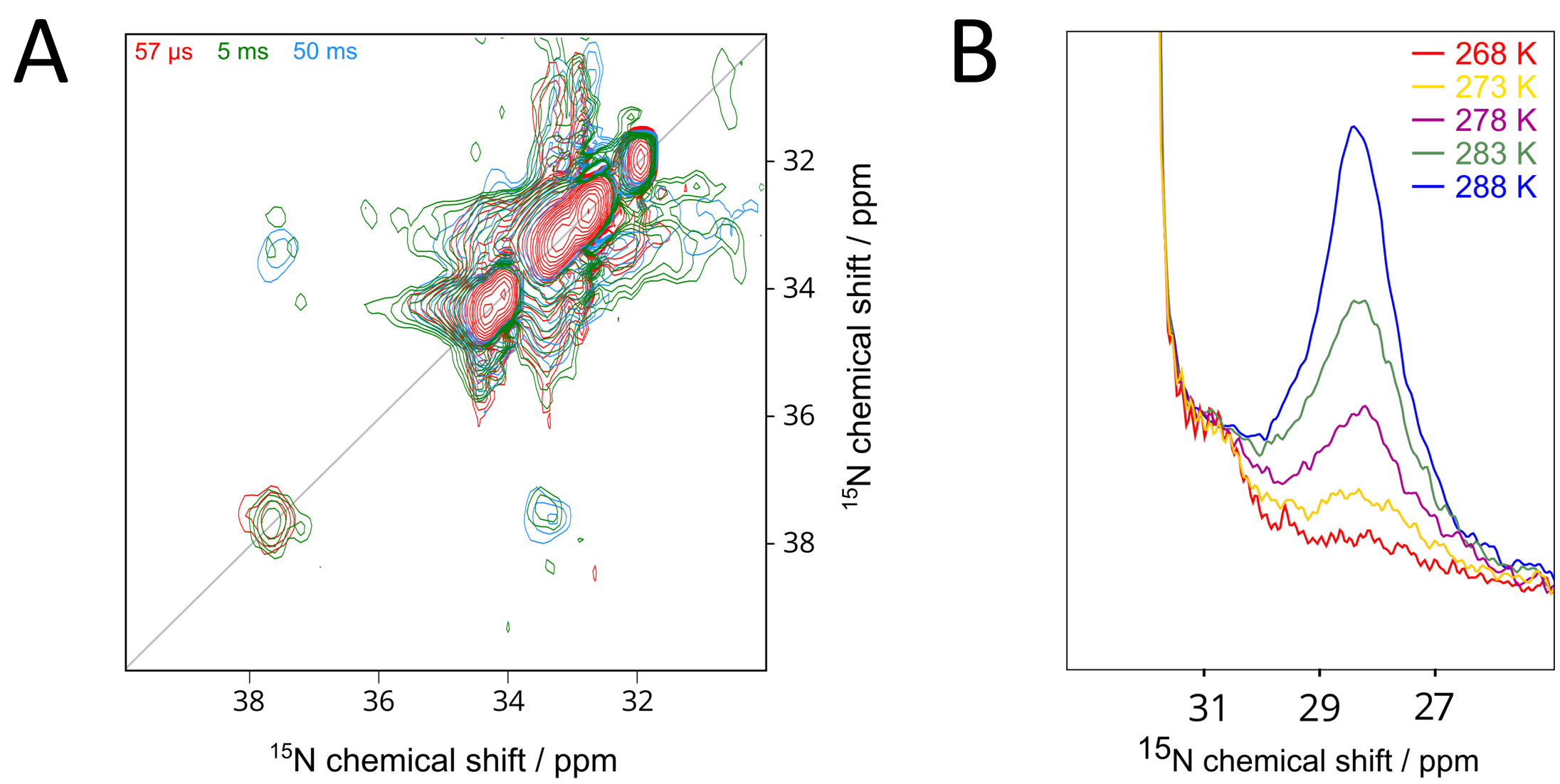

Fig. S10: (A) ^{15}N EXSY specta showing conformational exchange of Lys73 between two states with N^{ζ} chemical shifts of 37.5 ppm and 33.8 ppm; both shifts are consistent with a charged side chain. (B) Variable-temperature ^{15}N spectrum showing temperature-dependent narrowing of the Lys234 N^{ζ} resonance. Experimental data acquired at 600 MHz on a CPMAS CryoProbe at 13.5 kHz and 268 K.

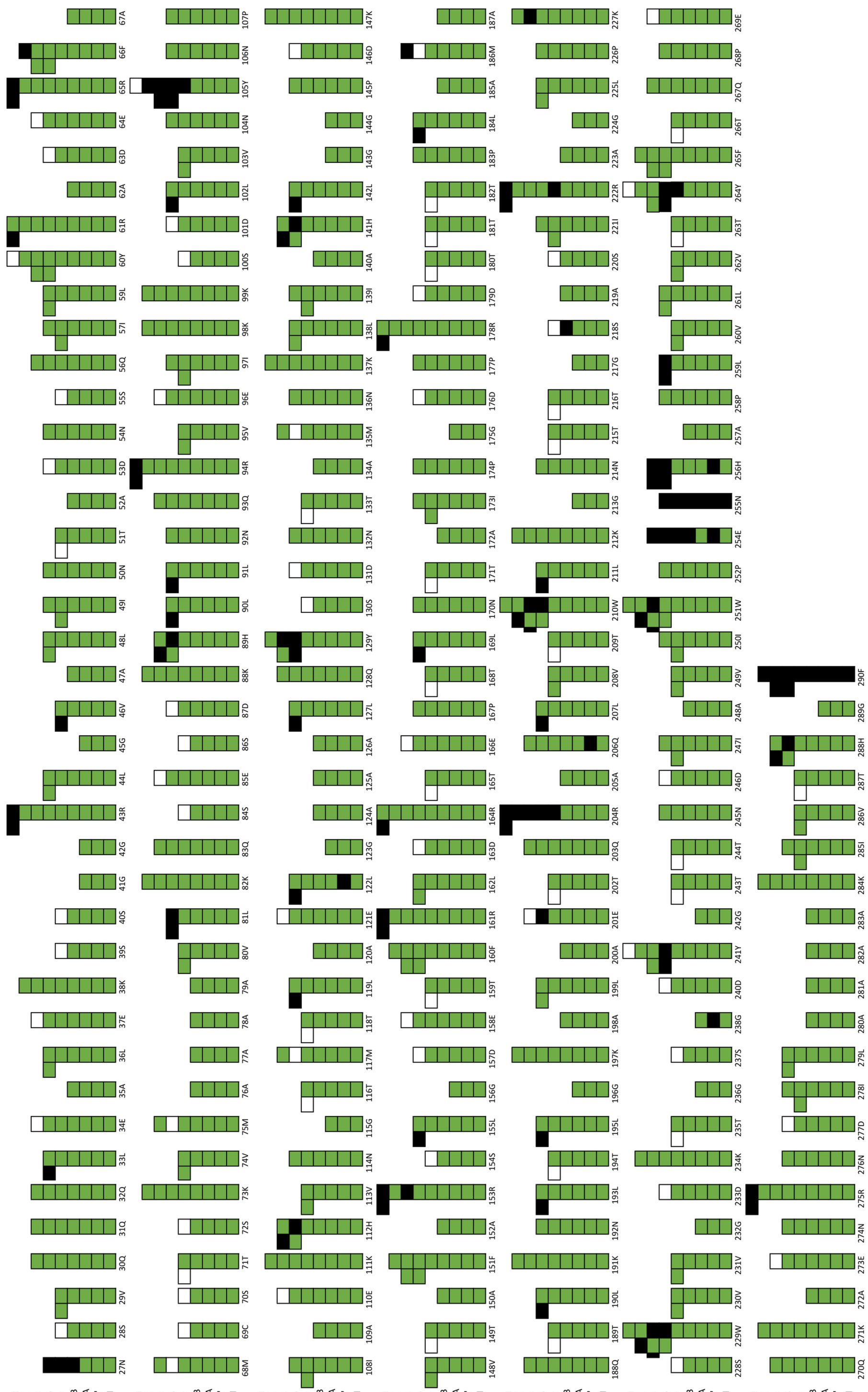

Fig. S11: Assignment completeness for Toho-1. Green and black boxes represent assigned and unassigned resonances respectively, while blank indicates a non NMR active nuclide. In total, 93% (1463/1572) of all carbon and nitrogen resonances were assigned, including 99.0% (772/780) of backbone atoms and 90.6% (637/703) of side-chain carbon atoms. By convention, the sequence for Toho-1 starts at residue 27 and ends, after 263 residues and three gaps (positions 58, 239, and 253), at residue 290.

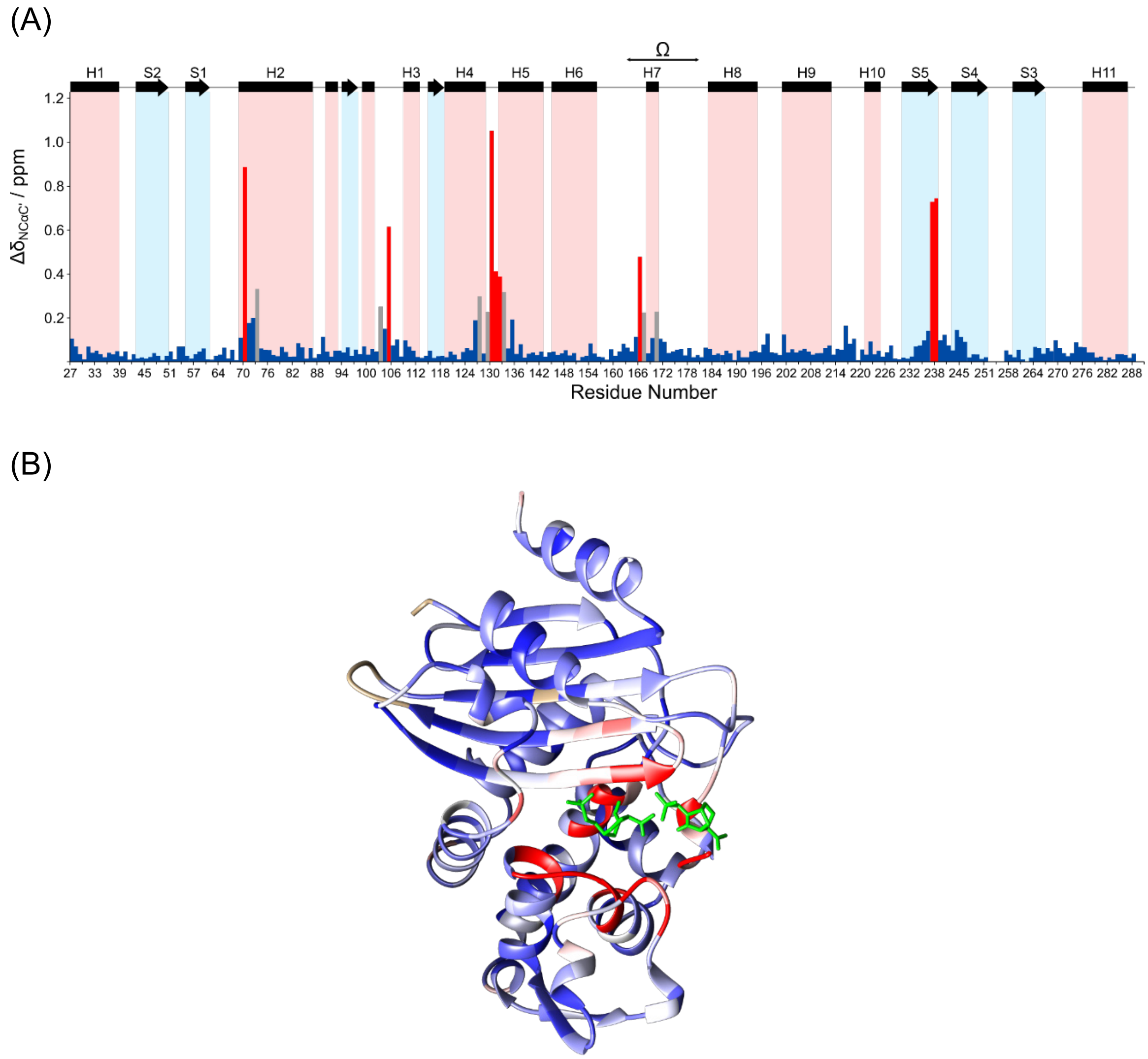

Fig. S12: (A) Residue-specific backbone chemical shift perturbations (CSPs) induced by avibactam binding to Toho-1, calculated as $\Delta\delta_{NC\alpha C'} = \sqrt{(0.15\Delta\delta_N)^2 + (0.28\Delta\delta_{C\alpha})^2 + (0.34\Delta\delta_{C'})^2}$ (47). Bars are colored according to CSP magnitude: blue, low; gray, moderate; and red, large. Secondary-structure elements from the Toho-1:avibactam crystal structure are indicated above the plot (H, α-helices; S, β-strands). (B) CSPs mapped onto the Toho-1:avibactam structure. Residues are colored on a continuous scale from blue (0 ppm) to white (mean CSP, 0.080 ppm) to red (mean + 1 SD, 0.211 ppm); residues lacking CSP data are shown in tan. Bound avibactam molecules are shown as green sticks.

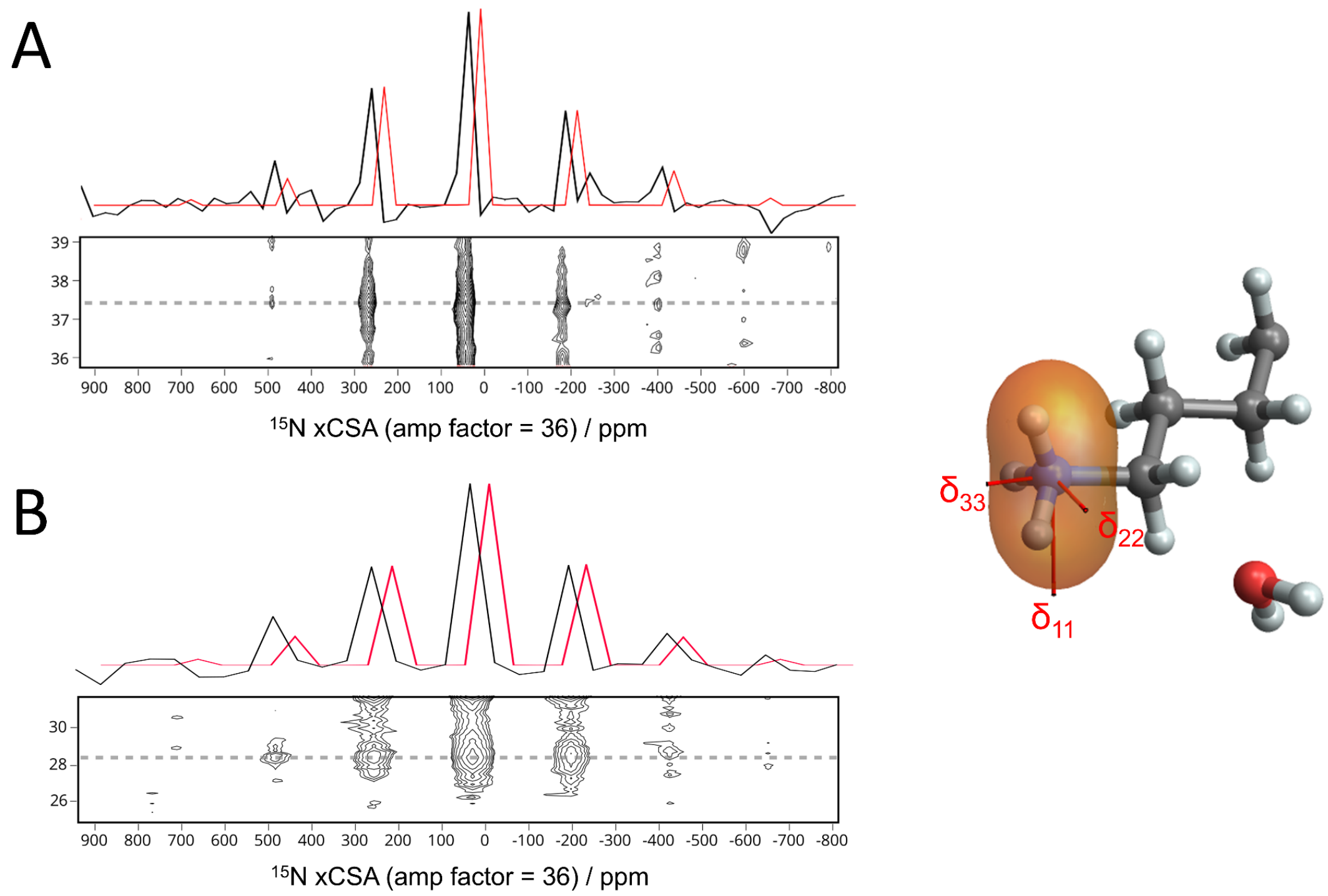

Fig. S13: ^{15}N Chemical shift tensors for N^{ζ} of (A) Lys73 and (B) Lys234 measured using the CSA amplification experiment with an amplification factor of 36 (1, 2). The fit (red) to the sideband manifold (5) in BrukerTopspin 4.1 allows for the extraction of the CSA principal axis components (A) (δ_{11}, δ_{22}, δ_{33}) = (50.1±0.8, 39.0±1.1, 23.4±0.6) ppm and (B) (δ_{11}, δ_{22}, δ_{33}) = (41.9±0.5, 28.3±1.1, 15.0±0.5) ppm. Accompanying molecule and tensor rendered in TensorView (6). Experimental data acquired at 600 MHz (^{1}H) on a CPMAS CryoProbe at 13.589 kHz and 268 K (A) and 288 K (B). Errors were determined by a numerical calculation of the covariance matrix and are reported as ±1 standard error.

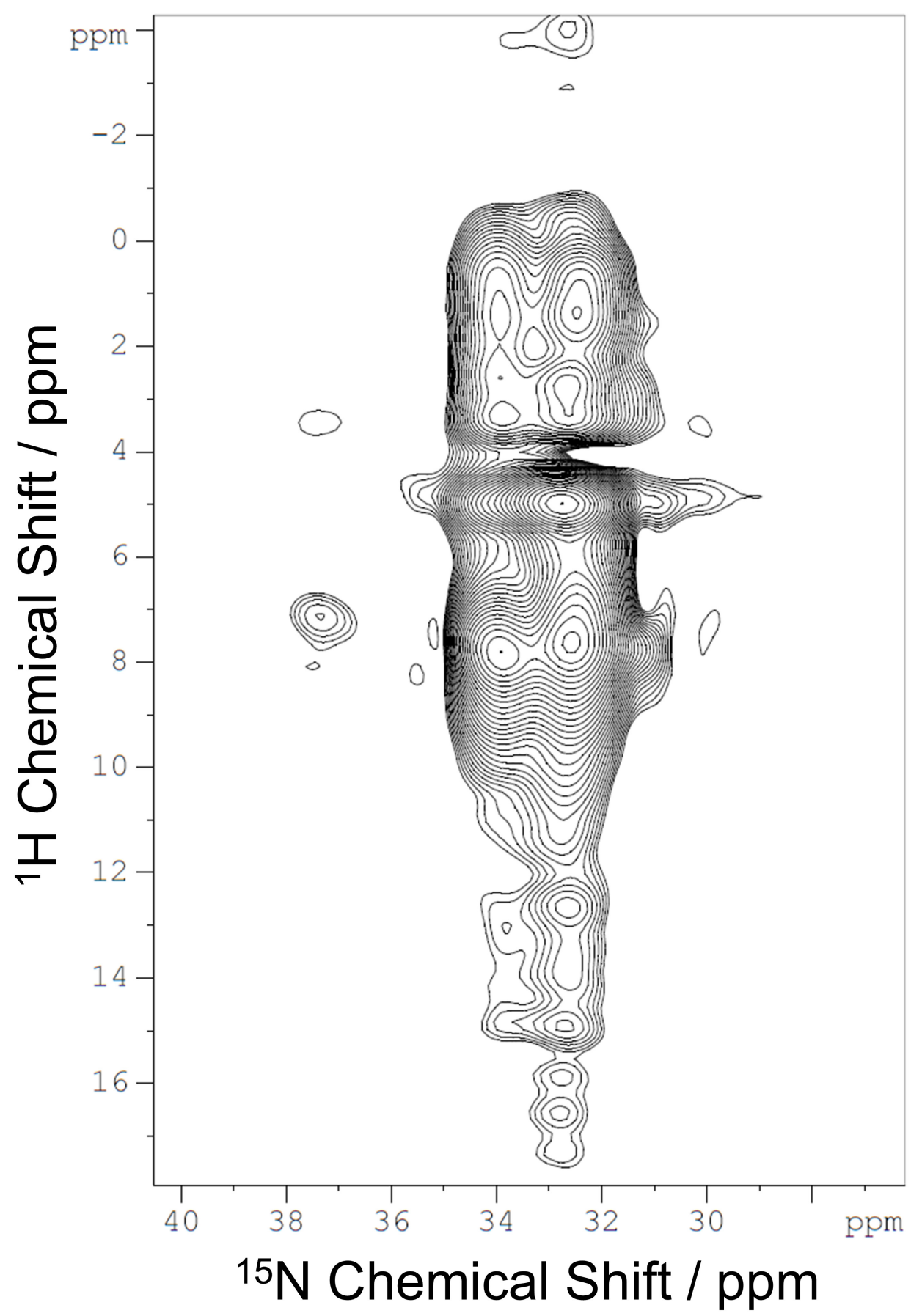

Fig. S14: ^{1}H-^{15}N FSLG-HETCOR (4) of the U-^{13}C,^{15}N-Lys Toho-1:avibactam complex shows a cross peak at 7.2 ppm ^{1}H and 37.5 ppm ^{15}N for the amino group of Lys73. Data acquired at 600 MHz (^{1}H) on a CPMAS CryoProbe at 13.5 kHz MAS and 268 K.

Table S6. Key experimental and first-principles isotropic chemical shifts and tensor principal components (PC) along with UC Model Probabilities (P_{UC}) for all 16 protonation state models

A. Isotropic Chemical Shifts [in ppm, relative to DSS (^{13}C) and liquid ammonia (^{15}N)]

Residue	Atom	Expt	Model 000	Model 001	Model 010	Model 011	Model 00’0	Model 00’1	Model 0’0’0	Model 0’0’1
Ser70	C^{β}	67.6	67.8	68.1	68.8	69.4	68.1	68.6	67.2	67.9
Lys73	C^{γ}	25.6	25.6	25.8	24.6	25	25.5	25.8	25.6	25.5
	C^{δ}	31.5	35.4	34.9	31.6	30.6	37.6	37.1	37.6	37.1
	C^{ε}	43.5	45.0	45.4	43.6	44.3	46.3	46.9	46.1	47.0
	N^{ζ}	37.5	30.3	29.1	37.5	37.1	23.5	22.8	23.5	22.6
Ser130	C^{β}	62.7	61.4	61.5	62.2	62.5	62.7	63.1	61.4	61.5
Asn132	C^{γ}	172.3	170.9	172.1	172.5	175	169.2	172.2	168.9	171.8
	N^{δ}	109.9	108	111.6	110.6	117.2	103.3	111.3	102.9	110.8
Glu166	C^{γ}	30.5	33.7	30.4	33.4	30.2	34.2	30.5	34.2	30.8
	C^{δ}	179.1	179.5	182	180.4	182.2	179.8	182.2	179.9	182.3
Asn170	C^{γ}	176.8	172.6	175.3	174.3	176.1	173.8	175.6	173.5	175.3
	N^{δ}	118	120.2	118.2	118.5	118.3	119.5	118.0	119.3	117.8
Lys234	C^{ε}	42.4	39.9	39.9	40.2	40.4	39.7	39.8	40.6	40.9
	N^{ζ}	28.4	14.1	14.1	14.5	14.4	14.7	14.4	15.6	15.7
		red-χ^2	4.45	3.23	2.24	2.70	6.92	5.46	6.85	5.26
		P_{UC}	0.00	0.01	0.04	0.02	0.00	0.00	0.00	0.00

Residue	Atom	Expt	0’10	0’11	100	101	110	111	10’0	10’1
Ser70	C^{β}	67.6	67.9	68.4	66.3	66.7	67.4	67.9	66.7	67.1
Lys73	C^{γ}	25.6	24.5	24.8	25	25.3	24.3	24.5	25	25.3
	C^{δ}	31.5	31.6	30.7	36.5	35.8	32.4	31	38.7	38
	C^{ε}	43.5	43.5	44.2	44.5	45.4	43.9	44.5	45.8	46.7
	N^{ζ}	37.5	38.0	37.5	34.2	31.5	36.9	36.2	25.3	24.1
Ser130	C^{β}	62.7	59.8	59.9	61.7	61.9	62.6	63.4	64	64.5
Asn132	C^{γ}	172.3	172.1	174.5	170.1	172.5	172.7	175.7	169.4	172.6
	N^{δ}	109.9	110.6	116.7	105.1	112.1	111.6	118.7	103.8	111.6
Glu166	C^{γ}	30.5	33.4	30.2	33.7	30.3	33.2	30.2	33.8	30.5
	C^{δ}	179.1	180.2	182.2	180.1	182	180.7	182.2	180.2	182.1
Asn170	C^{γ}	176.8	174.1	175.9	173.9	175.7	174.6	176.4	174.2	176
	N^{δ}	118.0	118.2	118.2	118.7	118.3	118.5	118.4	119.4	117.9
Lys234	C^{ε}	42.4	40.9	41.1	41.2	41.3	41.4	41.8	40.5	41.0
	N^{ζ}	28.4	16.1	16.1	25.7	26.4	26.8	26.0	25.8	26.1
		red-χ^2	2.40	2.48	3.40	2.22	1.10	1.83	6.28	4.82
		P_{UC}	0.03	0.03	0.01	0.04	0.72	0.10	0.00	0.00

Table S6. Key experimental and first-principles isotropic chemical shifts and tensor principal components (PC) along with UC Model Probabilities (P_{UC}) for all 16 protonation state models

B. Tensor Principal Components [in ppm, relative to DSS (^{13}C) and liquid ammonia (^{15}N)]

Residue	PC	Expt	Model 000	Model 001	Model 010	Model 011	Model 00'0	Model 00'1	Model 0'0'0	Model 0'0'1
Lys73	δ_{11}	50.1	57.8	57.0	49.7	48.6	53.1	52.4	52.7	52.6
N^{ζ}	δ_{22}	39.0	19.1	17.7	37.1	37.4	11.7	10.6	12.1	9.8
	δ_{33}	23.4	13.9	12.5	25.8	25.2	5.9	5.5	5.9	5.5
Glu166	δ_{11}	246.1	249.9	264.0	248.0	265.2	248.1	264.5	248.2	264.3
C^{δ}	δ_{22}	179.4	182.5	177.8	187.0	176.6	185.4	177.8	185.7	178.3
	δ_{33}	111.9	106.3	104.2	106.3	105	105.9	104.2	105.9	104.4
Lys234	δ_{11}	41.9	33.4	33.6	34.5	34.8	33.5	33.7	31.2	31.6
N^{ζ}	δ_{22}	28.3	6.3	6.4	7.1	7.1	7.0	6.6	12.4	12.4
	δ_{33}	15.0	2.6	2.3	1.9	1.4	3.6	3	3.2	3.2
		red-χ^2	2.52	4.73	1.77	3.86	3.46	5.67	3.18	5.36

Residue	PC	Expt	0'10	0'11	100	101	110	111	10'0	10'1
Lys73	δ_{11}	50.1	49.3	48.7	58.8	57.9	48.3	46.5	53.6	53.3
N^{ζ}	δ_{22}	39.0	36.9	37.2	22.5	19.5	38.1	38.1	15.8	12.9
	δ_{33}	23.4	27.8	26.7	21.2	17.2	24.4	23.8	6.6	6.2
Glu166	δ_{11}	246.1	247.9	264.8	248.0	264.2	247.9	265.5	247.9	264.5
C^{δ}	δ_{22}	179.4	186.5	176.8	186.2	177.3	188	176	186.8	177.2
	δ_{33}	111.9	106.3	105	106.2	104.5	106.4	105.2	106.0	104.5
Lys234	δ_{11}	41.9	30.2	30.2	38.7	39.1	39.9	37.8	36.6	37.1
N^{ζ}	δ_{22}	28.3	12.4	12.6	22.2	23.1	22.8	21.6	20.9	21.0
	δ_{33}	15.0	5.6	5.6	16.1	17.1	17.8	18.6	20.0	20.3
		red-χ^2	1.41	3.39	1.22	3.35	0.76	2.88	2.22	4.41

C. Composite Reduced-χ^2: Isotropic and Tensor Principal Components

	Model 000	Model 001	Model 010	Model 011	Model 00'0	Model 00'1	Model 0'0'0	Model 0'0'1
Composite red-χ^2	3.7	3.82	2.06	3.16	5.57	5.54	5.42	5.30
UC Model Probability P_{UC}	0.00	0.00	0.02	0.00	0.00	0.00	0.00	0.00

	0'10	0'11	100	101	110	111	10'0	10'1
Composite red-χ^2	2.01	2.84	2.55	2.66	0.97	2.24	4.69	4.66
UC Model Probability P_{UC}	0.02	0.00	0.01	0.00	0.94	0.01	0.00	0.00

SI References

1. I. Hung, Z. H. Gan, An efficient amplification pulse sequence for measuring chemical shift anisotropy under fast magic-angle spinning. *J Magn Reson* **213**, 196-199 (2011).
2. I. Hung *et al.*, Measuring $^{13}C/^{15}N$ chemical shift anisotropy in [$^{13}C,^{15}N$] uniformly enriched proteins using CSA amplification. *Solid State Nucl Magn Reson* **72**, 96-103 (2015).
3. J. O. Struppe *et al.*, Long-observation-window band-selective homonuclear decoupling: Increased sensitivity and resolution in solid-state NMR spectroscopy of proteins. *J Magn Reson* **236**, 89-94 (2013).
4. B. J. van Rossum, H. Forster, H. J. M. de Groot, High-field and high-speed CP-MAS C-13 NMR heteronuclear dipolar-correlation spectroscopy of solids with frequency-switched Lee-Goldburg homonuclear decoupling. *J Magn Reson* **124**, 516-519 (1997).
5. J. Herzfeld, A. E. Berger, Sideband Intensities in NMR-Spectra of Samples Spinning at the Magic Angle. *J Chem Phys* **73**, 6021-6030 (1980).
6. R. P. Young *et al.*, TensorView: A software tool for displaying NMR tensors. *Magn Reson Chem* **57**, 211-223 (2019).
7. J. B. Holmes *et al.*, Imaging active site chemistry and protonation states: NMR crystallography of the tryptophan synthase α-aminoacrylate intermediate. *P Natl Acad Sci USA* **119**, e2109235119 (2022).
8. B. G. Caulkins *et al.*, NMR Crystallography of a Carbanionic Intermediate in Tryptophan Synthase: Chemical Structure, Tautomerization, and Reaction Specificity. *J Am Chem Soc* **138**, 15214-15226 (2016).
9. S. S. Gunaga, R. W. Schurko, S. T. Holmes, F. Mentink-Vigier, Accessible hybrid DFT-quality NMR crystallography via gas-phase machine learning interatomic potentials. *ChemRxiv preprint 10.26434/chemrxiv.15000992.v2,* DOI: 10.26434/chemrxiv.15000992/v2 (2026).
10. B. M. Wood *et al.*, UMA: A family of universal models for atoms. *arXiv preprint arXiv:2506.23971*, DOI: 10.48550/arXiv.2506.23971 (2025).
11. A. H. Larsen *et al.*, The atomic simulation environment-a Python library for working with atoms. *J Phys Condens Mat* **29**, 273002 (2017).
12. M. J. Frisch *et al.*, *Gaussian 16, Revision C.01* (Gaussian, Inc., Wallingford, CT, 2016).
13. S. Moon, D. A. Case, A comparison of quantum chemical models for calculating NMR shielding parameters in peptides: Mixed basis set and ONIOM methods combined with a complete basis set extrapolation. *J Comput Chem* **27**, 825-836 (2006).
14. J. D. Hartman, T. J. Neubauer, B. G. Caulkins, L. J. Mueller, G. J. Beran, Converging nuclear magnetic shielding calculations with respect to basis and system size in protein systems. *J Biomol NMR* **62**, 327-340 (2015).
15. J. D. Hartman, R. A. Kudla, G. M. Day, L. J. Mueller, G. J. Beran, Benchmark fragment-based ^{1}H, ^{13}C, ^{15}N and ^{17}O chemical shift predictions in molecular crystals. *Phys Chem Chem Phys* **18**, 21686-21709 (2016).
16. J. D. Hartman, J. K. Harper, Improving the accuracy of GIPAW chemical shielding calculations with cluster and fragment corrections. *Solid State Nucl Magn Reson* **122**, 101832 (2022).
17. M. Dracínsky, P. Unzueta, G. J. O. Beran, Improving the accuracy of solid-state nuclear magnetic resonance chemical shift prediction with a simple molecular correction. *Phys Chem Chem Phys* **21**, 14992-15000 (2019).
18. J. D. Hartman, G. J. Beran, Accurate ^{13}C and ^{15}N molecular crystal chemical shielding tensors from fragment-based electronic structure theory. *Solid State Nucl Magn Reson* **96**, 10-18 (2018).
19. L. J. Mueller, Uniform chi-squared model probabilities in NMR crystallography. *Faraday Discuss* **255**, 203-221 (2025).
20. L. J. Mueller, "Quantitative Tools for Structure Selection in NMR Crystallography" in *Modern NMR Crystallography: Concepts and Applications,* D. L. Bryce, Ed. (Royal Society of Chemistry, 2025), chap. 5, pp. 96-121.

21. S. E. Ashbrook *et al.*, Challenges and opportunities for NMR calculations: general discussion. *Faraday Discuss* **255**, 288-310 (2025).
22. E. A. Engel *et al.*, A Bayesian approach to NMR crystal structure determination. *Phys Chem Chem Phys* **21**, 23385-23400 (2019).
23. A. Hofstetter, L. Emsley, Positional Variance in NMR Crystallography. *J Am Chem Soc* **139**, 2573-2576 (2017).
24. L. J. Mueller, Tensors and Rotations in NMR. *Concept Magn Reson Part A* **38a**, 221-235 (2011).
25. C. G. Williams *et al.*, Backbone assignment of a 28.5 kDa class A extended spectrum β-lactamase by high-field, carbon-detected solid-state NMR. *Biomol NMR Assign* **19**, 245-254 (2025).
26. O. A. Pemberton *et al.*, Mechanism of proton transfer in class A β-lactamase catalysis and inhibition by avibactam. *P Natl Acad Sci USA* **117**, 5818-5825 (2020).
27. S. J. Tomanicek *et al.*, Neutron Diffraction Studies of a Class A β-Lactamase Toho-1 E166A/R274N/R276N Triple Mutant. *J Mol Biol* **396**, 1070-1080 (2010).
28. S. J. Tomanicek *et al.*, The active site protonation states of perdeuterated Toho-1 β-lactamase determined by neutron diffraction support a role for Glu166 as the general base in acylation. *FEBS Lett* **585**, 364-368 (2011).
29. V. G. Vandavasi *et al.*, Exploring the mechanism of β-lactam ring protonation in the class A β-lactamase acylation mechanism using neutron and X-ray crystallography. *J Med Chem* **59**, 474-479 (2016).
30. P. S. Langan *et al.*, The structure of Toho1 β-lactamase in complex with penicillin reveals the role of Tyr105 in substrate recognition. *FEBS Open Bio* **6**, 1170-1177 (2016).
31. W. Kabsch, xds. *Acta Crystallogr D Biol Crystallogr* **66**, 125-132 (2010).
32. M. D. Winn *et al.*, Overview of the CCP4 suite and current developments. *Acta Crystallogr D Biol Crystallogr* **67**, 235-242 (2011).
33. P. D. Adams *et al.*, PHENIX: a comprehensive Python-based system for macromolecular structure solution. *Acta Crystallogr D Biol Crystallogr* **66**, 213-221 (2010).
34. P. Emsley, K. Cowtan, Coot: model-building tools for molecular graphics. *Acta Crystallogr D Biol Crystallogr* **60**, 2126-2132 (2004).
35. R. K. Harris *et al.*, Further conventions for NMR shielding and chemical shifts (IUPAC recommendations 2008). *Pure Appl Chem* **80**, 59-84 (2008).
36. C. R. Morcombe, K. W. Zilm, Chemical shift referencing in MAS solid state NMR. *J Magn Reson* **162**, 479-486 (2003).
37. G. Metz, X. L. Wu, S. O. Smith, Ramped-Amplitude Cross-Polarization in Magic-Angle-Spinning NMR. *J Magn Reson A* **110**, 219-227 (1994).
38. M. Baldus, A. T. Petkova, J. Herzfeld, R. G. Griffin, Cross polarization in the tilted frame: assignment and spectral simplification in heteronuclear spin systems. *Mol Phys* **95**, 1197-1207 (1998).
39. K. Takegoshi, S. Nakamura, T. Terao, ^{13}C-^{1}H dipolar-assisted rotational resonance in magic-angle spinning NMR. *Chem Phys Lett* **344**, 631-637 (2001).
40. G. Comellas, J. J. Lopez, A. J. Nieuwkoop, L. R. Lemkau, C. M. Rienstra, Straightforward, effective calibration of SPINAL-64 decoupling results in the enhancement of sensitivity and resolution of biomolecular solid-state NMR. *J Magn Reson* **209**, 131-135 (2011).
41. Y. Li, B. J. Wylie, C. M. Rienstra, Selective refocusing pulses in magic-angle spinning NMR: Characterization and applications to multi-dimensional protein spectroscopy. *J Magn Reson* **179**, 206-216 (2006).
42. C. Yang *et al.*, Crystal structure of the meta-stable intermediate in the photomechanical, crystal-to-crystal reaction of 9-butyl anthracene ester. *CrystEngComm* **18**, 7319-7329 (2016).
43. J. Cavanagh, W. J. Fairbrother, A. G. Palmer III, M. Rance, J. N. Skelton, *Protein NMR Spectroscopy: Principles and Practice* (Academic Press, ed. 2, 2007).
44. J. Schleucher *et al.*, A general enhancement scheme in heteronuclear multidimensional NMR employing pulsed field gradients. *J Biomol NMR* **4**, 301-306 (1994).

45. L. Kay, P. Keifer, T. Saarinen, Pure absorption gradient enhanced heteronuclear single quantum correlation spectroscopy with improved sensitivity. *J Am Chem Soc* **114**, 10663-10665 (1992).
46. L. E. Kay, G.-Y. Xu, A. U. Singer, D. R. Muhandiram, J. D. Forman-Kay, A gradient-enhanced HCCH-TOCSY experiment for recording side-chain ^1H and ^{13}C correlations in H_2O samples of proteins. *J Magn Reson B* **101**, 333-337 (1993).
47. F. H. Schumann *et al.*, Combined chemical shift changes and amino acid specific chemical shift mapping of protein-protein interactions. *J Biomol NMR* **39**, 275-289 (2007).
